# Proteins with an *Euonymus *lectin-like domain are ubiquitous in Embryophyta

**DOI:** 10.1186/1471-2229-9-136

**Published:** 2009-11-23

**Authors:** Elke Fouquaert, Willy J Peumans, Tom TM Vandekerckhove, Maté Ongenaert, Els JM Van Damme

**Affiliations:** 1 Laboratory of Biochemistry and Glycobiology, Department of Molecular Biotechnology, Ghent University, Coupure Links 653, 9000 Ghent, Belgium; 2 Laboratory of Bioinformatics and Computational Genomics, Department of Molecular Biotechnology, Ghent University, Coupure Links 653, 9000 Ghent, Belgium

## Abstract

**Background:**

Cloning of the *Euonymus *lectin led to the discovery of a novel domain that also occurs in some stress-induced plant proteins. The distribution and the diversity of proteins with an *Euonymus *lectin (EUL) domain were investigated using detailed analysis of sequences in publicly accessible genome and transcriptome databases.

**Results:**

Comprehensive in silico analyses indicate that the recently identified *Euonymus europaeus *lectin domain represents a conserved structural unit of a novel family of putative carbohydrate-binding proteins, which will further be referred to as the *Euonymus *lectin (EUL) family. The EUL domain is widespread among plants. Analysis of retrieved sequences revealed that some sequences consist of a single EUL domain linked to an unrelated N-terminal domain whereas others comprise two in tandem arrayed EUL domains. A new classification system for these lectins is proposed based on the overall domain architecture. Evolutionary relationships among the sequences with EUL domains are discussed.

**Conclusion:**

The identification of the EUL family provides the first evidence for the occurrence in terrestrial plants of a highly conserved plant specific domain. The widespread distribution of the EUL domain strikingly contrasts the more limited or even narrow distribution of most other lectin domains found in plants. The apparent omnipresence of the EUL domain is indicative for a universal role of this lectin domain in plants. Although there is unambiguous evidence that several EUL domains possess carbohydrate-binding activity further research is required to corroborate the carbohydrate-binding properties of different members of the EUL family.

## Background

Biochemical and molecular studies amply demonstrated that plants express a multitude of carbohydrate-binding proteins (also called lectins or agglutinins) [1,2]. Though a large number of these lectins have been studied in great detail at the biochemical, molecular, structural and physiological level, it is still not clear why plants accumulate proteins with no other obvious activity than reversibly binding to simple or complex glycans. For a long time plant lectins were regarded as a group of abundantly expressed proteins that are located in the vacuolar/extracellular compartment and preferentially bind to non-plant glycans. Accordingly, the concept was developed that most plant lectins do not interact with endogenous carbohydrates but function in the interaction with foreign organisms either in recognition or in defence-related phenomena [3,4]. Lectins that accumulate at (very) high levels in seeds or vegetative storage organs combine a function as a storage protein with a role in defence against phytophagous invertebrates or herbivorous animals [1,2,4].

Though applicable to the majority of all previously studied plant lectins, novel concepts had to be developed after the identification of several novel hormone or stress-responsive lectins. By virtue of their subcellular location and specificity this new class of lectins is at least in principle capable of interacting with endogenous receptors in the cytoplasmic/nuclear compartment of the plant cell [2,5-7]. Based on a comprehensive analysis of the data generated by biochemical, molecular biological and plant physiological studies, and genome/transcriptome/proteome surveys it was proposed recently that plants also express lectins that mediate specific protein-carbohydrate interactions in the cytoplasm and nucleus of the plant cell, and by doing so might play an important role in regulatory processes and/or cell signalling [8,9]. Meanwhile, evidence was reported that the jasmonate-induced tobacco leaf lectin, which is definitely located in the cytoplasm and nucleus, can interact *in situ *with conspecific N-glycosylated nuclear proteins [10]. Even in the absence of further insights into the mode of action, the latter findings put the physiological role of plant lectins in a new perspective because they indicate that at least some plant lectins interact - like many animal lectins - with endogenous glycan receptors [11-14]. However, it is still precocious to attribute an essential endogenous role to any of the currently known cytoplasmic/nuclear plant lectins until it is demonstrated that orthologs/homologs are ubiquitous among higher plants. Hitherto, five families of such inducible nucleocytoplasmic lectins have been identified [9].

Here we report the identification and *in silico *analysis of the family of cytoplasmic/nuclear protein(s) comprising domain(s) equivalent to the recently cloned *Euonymus europaeus *agglutinin [15]. The main objective of this research is to elaborate a comprehensive overview of the occurrence and evolution of this family of nucleocytoplasmic lectins and develop a unified classification system for this large and heterogeneous protein family. Our results show that proteins with (an) EUL (***Eu****onymus ***l**ectin) domain(s) are expressed in all Embryophyta - ranging from liverworts to flowering plants - for which a reasonable number of sequences has been deposited, but could not be found in any other eukaryote or prokaryote hitherto. Despite the EUL domain itself being fairly well conserved, the holoproteins comprising such (a) domain(s) exhibit a marked structural heterogeneity. Some proteins consist of a single EUL domain linked to an unrelated N-terminal domain whereas others comprise two in tandem arrayed EUL domains. Both the N-terminal domain and the linker sequence are highly variable. Transcriptome/genome analyses revealed that some species express a single EUL per diploid genome whereas up to eight structurally different proteins are found in others. Furthermore, expression analyses revealed that EUL domains are present in many stress response proteins suggesting a role of this lectin domain in stress signalling. The identification of the EUL family is discussed in view of the increasing importance of glycobiology in plant cell biology in general, and the understanding of the physiological role of plant lectins in particular.

## Results

### The *Euonymus *lectin domain represents the structural unit for a novel lectin family ubiquitous in terrestrial plants (Embryophyta)

A recent reinvestigation using a molecular approach revealed that the *Euonymus europaeus *agglutinin (EEA) cannot be assigned to one of the existing lectin families [15] but shares a high sequence identity/similarity (46%/62%) with a domain that was originally identified in two abscissic acid (ABA) and salt stress responsive rice proteins [16]. Based on the apparent Mr (in a 2-D gel) these rice proteins were called "OSR40 proteins". Though annotated in protein/gene databases as 'Ricin B related lectin domain containing proteins' detailed BLASTp, and PHI-BLAST and PSI-BLAST revealed that the OSR40 proteins share no decisive sequence similarity with any protein comprising a ricin B domain but undoubtedly belong to the same family as the *Euonymus *agglutinin [15] (Additional file 1: Figures S1A and S1B). A BLASTp search of the NCBInr protein database using the sequence of EEA as a query yielded a set of 120 entries with E-value <1^e-05^. Due to redundant annotations the number of (putative) proteins is considerably lower (approximately 50). Of all these entries only EEA has been purified and characterized. For a few others (like the rice OSR40 proteins) there is experimental evidence based on protein analysis techniques that they are actually expressed. All other hits detected by BLASTp searches refer to hypothetical proteins the sequence of which is deduced from either cDNA or genomic sequences. At present there is no uniform naming for all these putative proteins. Most of them are still annotated as "putative/hypothetical protein", "expressed" protein, "unknown" protein or "stress-responsive" protein.

Several experimental data unambiguously demonstrate that the EUL domain represents a new carbohydrate-binding domain. First, glycan array binding studies showed that EEA has high affinity towards blood group B oligosaccharides, but also binds to high mannose N-glycans [15]. Second, Edman degradation of a previously characterized lectin from tulip (*Tulipa gesneriana*) bulbs (called TxLMI) [17] that until now could not be classified in one of the known plant lectin families revealed that the N-terminus of the 19 kDa subunits shares >66% sequence identity with EUL proteins from other monocots (Additional file 1: Figure S1C). Additional sequences of tryptic peptides confirmed that TxLMI comprises a typical EUL domain (Additional file 1: Figure S1C). Third, preliminary experiments revealed that the EUL homolog from *Arabidopsis thaliana *(At2 g39050) expressed in *Pichia pastoris *agglutinates rabbit erythrocytes (J. Van Hove and E. Van Damme, unpublished data) and hence must be capable of interacting with carbohydrate structures present in the erythrocyte membrane. The obvious carbohydrate-binding activity of three different EUL proteins from taxonomically unrelated species strongly indicates that several EUL domains can be considered lectin domains. It should be emphasized, however, that sequence similarity to the *Euonymus *lectin does not necessarily imply that a given domain possesses carbohydrate-binding activity.

Transcriptome analyses showed that virtually all Embryophyta species for which a reasonable number of sequences were deposited express one or more proteins with a domain similar to the EEA polypeptide (see below). Therefore, it appears that the *Euonymus *lectin domain represents the structural unit for a novel lectin family ubiquitous in terrestrial plants (Embryophyta). Since neither the rice OSR40 proteins nor the putative homologs found in other plants can be classified under the ricin B family, a more appropriate nomenclature should be introduced. Taking into account that the *Euonymus *agglutinin was the first identified member and, in addition, possesses a well-defined biological activity (*in casu *carbohydrate-binding), it seems logical to name this novel protein family after the *Euonymus *agglutinin and consider the *Euonymus *lectin domain as the diagnostic structural unit. Accordingly, the term '*Euonymus *lectin domain' (or 'EUL domain') will be adopted for all structural units equivalent to the *Euonymus *lectin subunit, and the term 'EUL family' will be used to refer to the group of proteins containing at least one EUL domain.

To the best of our knowledge the acronym EUL is not used yet for any protein family or conserved protein domain, and hence can be introduced without the risk of confusion. The terms 'EUL' and 'EUL family' are preferred to 'OSR40' and 'OSR40 proteins' for two reasons. First, the name 'OSR40' includes no information about the biological activity of the domain. Second, the term 'OSR40' does not refer to a well-defined structural unit but to a 40 kDa ABA/salt stress responsive rice protein with a complex structure (encompassing two homologous domains of approximately 150 AA residues separated by a linker sequence plus an extra N-terminal domain). Hence, the term 'OSR40' is inappropriate to refer to all related proteins that include only a single 150 AA domain and differ in molecular mass.

### Domain architecture and nomenclature

**Figure 1:**
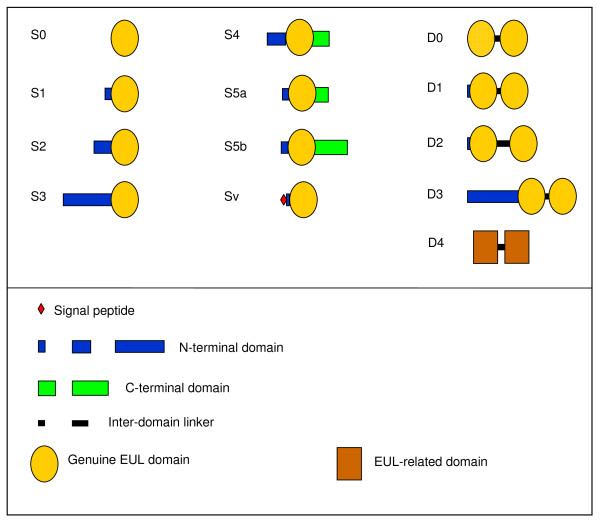
**Schematic representation of identified EULarchitectures found in Embryophyta**. Some putative EUL proteins comprise one EUL domain, while others comprise two in tandem arrayed EUL domains. In most EUL proteins the EUL domain is preceded by an unrelated N-terminal domain varying in length. In a few EUL proteins a signal peptide was detected at the N-terminus. Some EUL proteins comprise a C-terminal domain which can also differ in length. In two-domain EUL proteins the two domains are separated by a linker.

Comprehensive sequence analyses demonstrated marked differences in the overall structure of proteins with EUL domain(s). Based on the sequence information available at present, seven types of proteins containing a single EUL domain and five types of proteins with two EUL domains can be distinguished (Figure 1). Table 1 summarizes the different types of EULs and examples of plants in which such proteins can be found. Basically, the EUL family can be subdivided into single- and two-domain proteins. Some single-domain proteins consist - like the *Euonymus *lectin - exclusively of a sole EUL domain. However, in most cases the EUL domain is preceded by an unrelated N-terminal domain varying in length and composition/sequence. In a minority of the single-domain EULs a signal peptide was detected at the N-terminus, indicating that some vacuolar EULs presumably also exist. In addition to an N-terminal domain, an extra domain at the C-terminus which can also differ in length is found in a few single-domain EULs. Most but not all two-domain proteins contain an extra N-terminal sequence and a linker between the two EUL domains. Despite differences in the length of the N-terminal sequences as well as the linkers, the subfamily of two-domain EULs is less heterogeneous than that of the single-domain proteins.

**Table 1:** Overview of the different types of EULs and their occurrence in plants.

Different types of EUL		Plant species
**Type S: Proteins with a single EUL domain**		

Type S0	Proteins consisting of an EUL domain only	*Euonymus europaeus*, *Plantago major*, *Zea mays*, *Selaginella moellendorfii*
Type S1	Proteins consisting of an EUL domain preceded by a short (<50 AA) unrelated N-terminal sequence	*Physcomitrella patens*, *Triticum aestivum*, *Hordeum vulgare*, *Selaginella moellendorfii*
Type S2	Proteins consisting of an EUL domain preceded by a medium long (50-100 AA) unrelated N-terminal sequence.	*Oryza sativa*, *Sorghum bicolor*, *Zea mays, Lactuca *sp., *Pinus taeda*, *Picea sitchensis*
Type S3	Proteins consisting of an EUL domain preceded by a long (>100 AA) unrelated N-terminal sequence.	*Arabidopsis thaliana*, *Medicago truncatula*, *Vitis vinifera*, *Populus trichocarpa*, *Oryza sativa*, *Sorghum bicolor*, ...
Type S4	Proteins consisting of a medium long (50-100 AA) unrelated N-terminal sequence, an EUL domain, and a short (<50) C-terminal extension.	*Selaginella moellendorfii*
Type S5a/S5b	Proteins consisting of a short (<50) unrelated N-terminal sequence, an EUL domain, and a short (<50) or medium long (50-100 AA) C-terminal extension.	*Marchantia polymorpha*
Type Sv	Proteins consisting of an EUL domain preceded by a short unrelated N-terminal sequence containing a signal peptide (vacuolar form of the EUL).	*Sorghum bicolor*, *Zea mays*, *Hordeum vulgare*, *Triticum aestivum*, *Selaginella moellendorfii*

**Type D: Proteins with two in tandem arrayed EUL domains**		

Type D0	Proteins consisting of two in tandem arrayed EUL domains separated by a short (<40 AA residues) linker and without N-terminal extension.	*Selaginella moellendorfii*
Type D1	Proteins consisting of two in tandem arrayed EUL domains separated by a short (<40 AA residues) linker and preceded by a short (15-35 AA residues) N-terminal extension	*Oryza sativa, Sorghum bicolor*, *Zea mays, Hordeum vulgare*, *Triticum aestivum, Pinus taeda*, *Picea sitchensis, Physcomitrella patens*
Type D2	Proteins consisting of two in tandem arrayed EUL domains separated by a long (>40 AA residues) linker and preceded by a short (10-35 AA residues) N-terminal extension	*Oryza sativa, Sorghum bicolor*, *Zea mays, Hordeum vulgare*, *Triticum aestivum, Pinus taeda*, *Picea sitchensis*,
Type D3	Proteins consisting of two in tandem arrayed EUL domains separated by a short (<40 AA residues) linker and preceded by a long (>50 AA residues) N-terminal extension	*Selaginella moellendorfii*
Type D4	Proteins consisting of two in tandem arrayed EUL-related domains separated by a short (<40 AA residues) linker and without N-terminal extension. Note that the domains of these proteins share only low sequence similarity with the genuine EUL domains.	*Selaginella moellendorfii*

A schematic overview of the different types is shown in Fig. 1.

The obvious differences in domain architecture between different members of the EUL family combined with the simultaneous occurrence of multiple structurally different EULs in some species necessitate a consistent nomenclature. Therefore, a classification system was elaborated based on the overall domain architecture and the length/sequence peculiarities of the accessory domains. Individual proteins containing an EUL domain are indicated by a species code, composed of the three first characters of the genus name and the first two characters of the species name, followed by one of the 12 domain architecture codes (Table 1). If in one species different forms of a given type occur, different subtypes and isoforms are then indicated by additional characters.

### Occurrence of plant proteins containing one or more EUL domains in sequenced genomes

**Figure 2:**
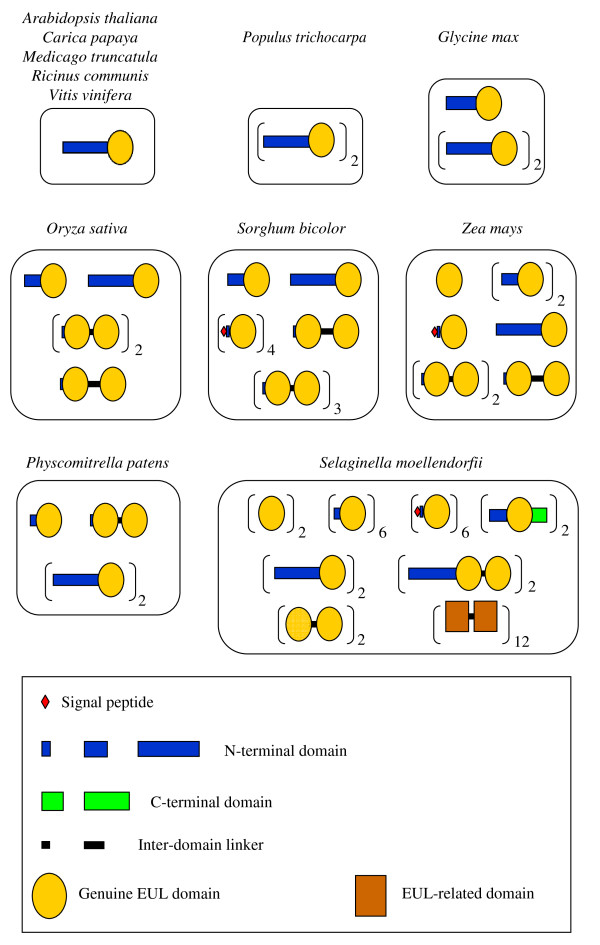
**Schematic overview of the different types of EUL proteins in plant species for which complete genome sequences are available**. Analyses were done for the genomes of *Arabidopsis thaliana, Carica papaya, Medicago truncatula, Ricinus communis, Vitis vinifera, Populus trichocarpa, Glycine max, Oryza sativa, Sorghum bicolor, Zea mays, Physcomitrella patens *and *Selaginella moellendorfii*. The number after the brackets indicates the number of copies found for one particular EUL architecture.

Initial screening of the plant databases unveiled two important aspects of the EUL family. First, this family comprises proteins with a markedly different overall structure. Second, different species express different sets of EULs. To unravel the complex set of data a comprehensive analysis was made of the EUL proteins/genes found in plants for which (nearly) complete genome sequences are available (Figure 2).

#### Dicot genomes with a single EUL gene: *Arabidopsis thaliana*, *Medicago truncatula*, *Vitis vinifera*, *Ricinus communis* and *Carica papaya*

The *Arabidopsis thaliana *genome harbours a single EUL gene/protein (At2 g39050). According to both the TIGR and TAIR annotations At2 g39050 belongs to a 'Hydroxyproline-rich glycoprotein family' and contains a 'QXW lectin repeat domain' (Pfam: PF00652 = Ricin-type beta-trefoil lectin domain), whereas in the MIPS database the protein is annotated as 'similar to stress responsive lectin-like cDNAs from rice'. However, it is evident now that the C-terminal part of this *Arabidopsis *protein corresponds to an EUL domain and accordingly should be classified in the EUL family. The 154 AA EUL domain in this *Arabidopsis *protein shows 44% sequence identity and 71% sequence similarity to the EUL domain of EEA. In addition, this protein contains a long (approximately 160 residues) unrelated N-terminal domain that shares no significant similarity with any other known domain. Due to the presence of this long N-terminal domain the *Arabidopsis *EUL is classified as a type S3 EUL (ArathEULS3).

Single orthologs of the *ArathEULS3 *gene were also identified in the completely sequenced genomes of *Medicago truncatula *(barrel clover), *Vitis vinifera *(common grape vine), *Ricinus communis *(castor bean) and *Carica papaya *(papaya). The corresponding proteins are expressed and have the same domain architecture as ArathEULS3 (Figure 2; Additional file 1: Figure S2).

#### Populus trichocarpa: dicot genome with two paralogous EUL genes

Two orthologs of the *Arabidopsis *EUL were identified in the *Populus trichocarpa *genome. The putative proteins PoptrEULS3A and PoptrEULS3B consist of a long N-terminal domain and an EUL domain, and are therefore also classified as S3 type EULs (Figure 2; Additional file 1: Figure S2). The two EUL proteins from poplarhave nearly identical EUL domains but differ by four deletions/insertions in their respective N-terminal domains. Since all identified ESTs (>20) apparently correspond to *PoptrEULS3A *there is some uncertainty about the expression of *PoptrEULS3B*.

#### Glycine max: dicot genome with three paralogous EUL genes

The genome of soybean comprises two genuine orthologs of the *Arabidopsis *EUL (GlymaEULS3A and GlymaEULS3B) that are located at different loci and according to transcriptome data are expressed. In addition, a third gene (GlymaEULS3C) tandemly arrayed to GlymaEULS3B could be identified that encodes an EUL protein with a shorter N-terminal domain (Figure 2). No corresponding ESTs or cDNAs could be retrieved in *Glycine max (*or any other legume). The occurrence of GlymaEULS3A and GlymaEULS3B can be explained by the fact that soybean is a "diploidized tetraploid", whereas GlymaEULS3C most probably results from an in tandem duplication. At present, no similar in tandem arrayed pair of EUL genes was identified in any other dicot. However, as described below, in tandem duplication made an important contribution to the evolution of EUL genes in grasses (and perhaps in other monocots as well).

#### Oryza sativa: monocot genome with a set of 5 expressed EUL proteins

In 1995, Moons *et al*. [18] identified a 40 kDa histidine-rich ABA-responsive protein (called OSR40c1) in rice roots. Sequencing of genomic fragments combined with Western blotting experiments using antisera raised against a conserved OSR40 peptide further proved that at least two other OSR40 proteins accumulated in roots of rice seedlings upon exposure to salt stress, namely OSR40 g2 and OSR40 g3 [16]. Therefore, it was concluded that the OSR40 proteins, which are now classified as EULs, belong to a multigene family.

BLAST searches against the completed rice genome confirmed the occurrence of an EUL gene family. Nine genes could be identified that encode proteins comprising one or two EUL domains. Expression was detected for only five of these genes (Figure 2) suggesting that four genes might be pseudogenes. The corresponding five proteins represent four different types of EULs: (i) a single-domain protein with a medium long unrelated N-terminal sequence (OrysaEULS2 = OSR40 g3), (ii) a single-domain EUL protein with a long unrelated N-terminal sequence (OrysaEULS3 = r40c1), (iii) two two-domain proteins with a short linker (OrysaEULD1A = OSR40 g2, and OrysaEULD1B = OSR40c1), and (iv) one two-domain protein with a long linker (OrysaEULD2 = OSR40c2).

Based on its overall domain structure OrysaEULS3 can be considered a genuine ortholog of the *Arabidopsis*-type EUL(s) (Additional file 1: Figure S2). The nine rice (pseudo)genes with EUL domains are located at four loci on four different chromosomes: *OrysaEULS3 *on chromosome 1; *OrysaEULS2, OrysaEULD1A *and *OrysaEULD2 *as a cluster on chromosome 7; *OrysaEULD1B*, clustered with the two non-expressed (pseudo)genes *OrysaEULS0A *and *OrysaEULD0*, on chromosome 3; the two non-expressed (pseudo)genes *OrysaEULS0B *and *OrysaEULS0C *as a cluster on chromosome 12.

Because no trace of type S0 EUL expression could be detected in *O. sativa*, it is suggested that the OrysaEULS0B and OrysaEULS0C genes might be pseudogenes in *O. sativa*. Nonetheless this conclusion cannot be extrapolated to all *Oryza *species. A cDNA sequence encoding an S0 type EUL was deposited, indeed, for *O. punctata*. Interestingly, a virtually identical nucleotide sequence can be assembled from the *O. sativa *genomic sequence by joining the first exon of Os12 g08340 and the second exon of Os12 g08310. A closer examination shows that the genomic sequences covering Os12 g08340 and Os12 g08310 contain the coding sequence of an S0 type EUL protein (as expressed in *O. punctata*) in which the exons encoding the N- and C-terminal part are interrupted by a very long intron (20,736 nucleotides) that apparently comprises a transposon. This might indicate that the *O. sativa *gene encoding a S0 type EUL protein was - in evolutionary terms - recently inactivated through the insertion of a transposon.

#### *Sorghum bicolor*: monocot genome with a complex set of 'cytoplasmic' and 'vacuolar' EUL proteins

BLAST searches in the genome and transcriptome databases indicated that *Sorghum bicolor *expresses closely related orthologs of all five EUL proteins expressed in *O. sativa *(i.e. SorbiEULS2, SorbiEULS3, SorbiEULD1A, SorbiEULD1B and SorbiEULD2) (Figure 2). In addition the *S. bicolor *genome contains also (expressed) EUL genes that are not found in the rice genome. First, there is a third two-domain protein (SorbiEULD1C) for which no ortholog could be identified in rice. Second, the genome apparently contains four genes (*SorbiSv1-4*) encoding single domain EUL proteins that are synthesized with a signal peptide. Though the exact subcellular location of these proteins is not known, it seems evident that they are synthesized in the ER and follow the secretory pathway. To distinguish them from the 'cytoplasmic' EUL they are referred to as 'vacuolar' EULs. For both *SorbiSv1 *and *SorbiSv2 *corresponding ESTs could be retrieved indicating that these genes are expressed.

#### *Zea mays*: monocot genome with a complex set of 'cytoplasmic' and 'vacuolar' EUL proteins

Analyses of genome and transcriptome databases confirmed that *Z. mays *expresses orthologs of all five rice EUL proteins (referred to as *ZeamaEULS2*, *ZeamaEULS3*, *ZeamaEULD1A*, *ZeamaEULD1B *and *ZeamaEULD2*) (Figure 2). In addition, maize expresses two very similar single-domain EUL proteins lacking an N-terminal domain (ZeamaEULS0a and ZeamaEULS0b) as well as a presumed vacuolar single-domain EUL protein (ZeamaSv). Corresponding genomic sequences are available for *ZeamaEULS0a *but not yet for *ZeamaEULS0*b and *ZeamaEULSv*.

#### *Physcomitrella patens*: a moss genome with a set of 3 single-domain and 1 two-domain EUL genes

From the *P. patens *databases it could be derived that this moss genome contains three genes encoding single-domain EULs *(PhypaEULS1*, *PhypaEULS3A*, and *PhypaEULS3B*) and a single gene encoding a two-domain EUL protein *(PhypaEULD1*) (Figure 2). Perfectly matching EST sequences were deposited for *PhypaEULS3A*, *PhypaEULS3B*, and *PhypaEULD1 *but not for *PhypaEULS1*, casting doubt on whether *PhypaEULS1 *is expressed.

#### *Selaginella moellendorffii*: a spike moss genome with a set of 5 single-domain and 3 two-domain EUL genes

Detailed analysis of genome and transcriptome databases of *Selaginella moellendorffii *(belonging to the Lycopodiophyta, the oldest vascular plant division) resulted in the identification of a complex set of at least 34 genes encoding EULs of eight different types: four different single-domain cytoplasmic types (SelmoEULS0, SelmoEULS1, SelmoEULS3, and SelmoEULS4), one single-domain vacuolar type (SelmoEULSv), and three two-domain types (SelmoEULD0, SelmoEULD3, and SelmoEULD4) (Figure 2). For each EUL a nearly identical paralog exists (e.g. SelmoS1Aa and SelmoS1Ab). It is worth mentioning that 4 of these eight types (namely SelmoEULS4, SelmoEULD0, SelmoEULD3, and SelmoEULD4) have not been found in any other plant species.

S4-type EULs resemble the S2- and S3-type proteins found in monocots and dicots but distinguish themselves by the presence of an extra 34 AA residue C-terminal domain. Moreover, the latter is located on a separate exon. Besides these "unique" S4-type genes the *S. moellendorffii *genome contains three "novel" types of two-domain genes. Two genes (*SelmoD0a-b*) encode two-domain proteins without N-terminal domain and two other *(SelmoD3a-b) *two-domain EULs with a long N-terminal domain. Finally, the *Selaginella *genome contains at least 6 pairs of genes (*SelmoD4A-Fa-b*) encoding SelmoEULD4 proteins. SelmoEULD4 proteins are like SelmoEULD0 two-domain EULs without N-terminal domain. However, they are only distantly related to other two-domain EULs. ESTs have been identified for all types of proteins (though not for all individual genes) except for SelmoEULS0, SelmoEULS1C, and for the vacuolar proteins.

### Additional data from transcriptome analyses

**Figure 3:**
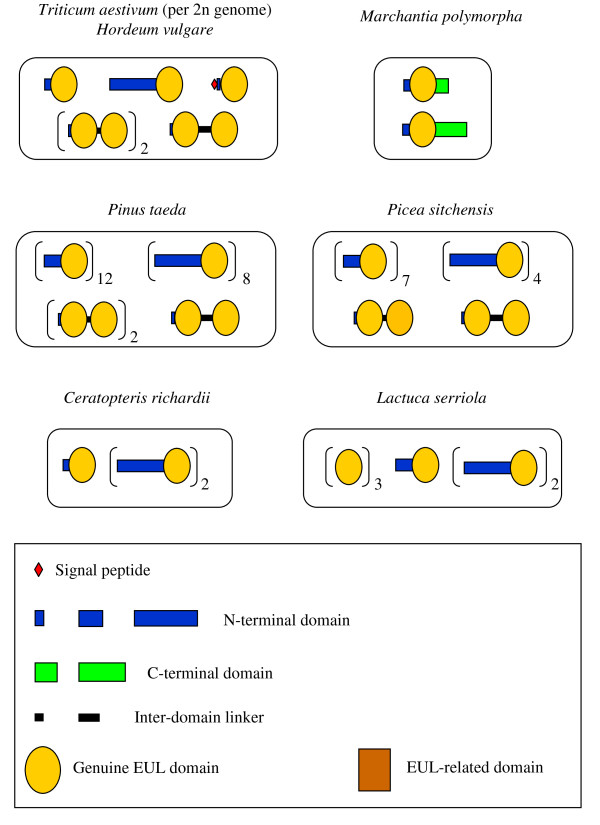
**Schematic overview of the different types of EUL proteins found in the transcriptome**. Analyses were done for *Triticum aestivum*, *Hordeum vulgare, Marchantia polymorpha, Pinus taeda, Picea sitchensis, Ceratopteris richardii*, and *Lactuca serriola*. The number after the brackets indicates the number of copies found for one particular EUL architecture.

To further corroborate the presence and composition of the EUL gene complement in other plant species, a thorough analysis was performed of available transcriptome data (Figure 3). A detailed discussion on the EUL sequences found in all major taxonomic groups is given in Additional file 2.

Interestingly, two single-domain EULs that have not been identified in any other plant species have been retrieved from the transcriptome of the liverwort *Marchantia polymorpha*. Both proteins comprise a short N-terminal domain followed by an EUL domain and a short to medium long C-terminal domain (Figure 3). EST encoding proteins with an EUL domain were also deposited for ferns (*Ceratopteris richardii *and *Adiantum capillus-veneris*) and cycads (*Cycas rumphii *and 3 *Zamia *species). Conifers such as *Pinus taeda *(loblolly pine) also express a complex set of EUL proteins comprising S2, S3, D1 and D2-type proteins. Although the complement of EUL proteins expressed in *Picea sitchensis *(Sitka spruce) resembles that in *P. taeda *it is certainly not identical (Figure 3).

Within flowering plants some ESTs encoding EUL proteins are found in basal Magnoliophyta (e.g. *Amborella trichopoda*) and Magnoliids (e.g. *Liriodendron tulipifera*). Within Eudicotyledons EUL sequences are present in stem eudicotyledons, (e.g. *Aquilegia formosa *× *Aquilegia pubescens*) as well as in virtually all EST databases from core eudicotyledons. Most species express a single S3-type protein per diploid genome but several species (e.g. *Lactuca *sp., *Helianthus *sp., *Antirrhinum majus*) express complex mixtures of S3-type EUL proteins.

A search for EUL domains in Liliopsida (monocotyledons) revealed that all EST databases from monocots contain sequences encoding EUL domains. The whole of retrieved sequences indicates that most if not all of these monocots express a set of single-domain and two-domain proteins comparable to that found in *O. sativa*.

### *In silico *expression analysis of the EUL from *Arabidopsis*

Several cDNA and EST sequences have been identified that confirm the expression of the EUL homolog ArathEULS3 (At2 g39050) in Arabidopsis. Therefore the expression profile of this EUL homolog was studied using the *Arabidopsis *electronic fluorescent protein browser [19].

The expression of ArathEULS3 is developmentally regulated with a high expression in senescent leaves and in flowers from the 15^th ^flower stage. The highest absolute fluorescence value in untreated plants (955) was observed in the sepals of flowers but cauline leaves also clearly show expression of ArathEULS3. Microarray expression analyses of leaf mesophyl cells and guard cells [20] revealed that ArathEULS3 is weakly expressed in the mesophyl cells of 5 week-old leaves (absolute value of 98.78), but is highly expressed in the guard cells (absolute value of 723.92). This expression in guard cells was increased more then 2-fold in leaves floated on 100 μM ABA (absolute value of 1633).

The relative expression (defined as the ratio between the absolute fluorescence values measured for a given tissue with and without treatment) of ArathEULS3 was studied for different abiotic as well as biotic stresses (Additional file 1: Figure S3). ArathEULS3 is upregulated 11-fold in shoots of 18 day-old plants floated on liquid Murashige and Skoog medium supplemented with 300 mM mannitol for 12 h. Similarly salt stress (150 mM NaCl) and drought stress cause an 8-fold and 2.5-fold upregulation, respectively, of ArathEULS3 expression after 24 h salt treatment and 3 h drought treatment (Additional file 1: Figure S3A). Other abiotic stress treatments such as oxidative stress, wounding, heat and UV treatment, and application of chemicals such as cycloheximide, brassinosteroid inhibitors, auxin inhibitors and gibberellic acid inhibitors do not affect the expression of ArathEULS3. In contrast, a treatment of seedlings with the plant hormone ABA resulted in a 7-fold upregulation of the gene, already after 3 h treatment. Similarly a treatment with methyl jasmonate resulted in a 2.5-fold upregulation of ArathEULS3 after 3 h treatment (Additional file 1: Figure S3B).

Next to abiotic stresses, ArathEULS3 gene expression was also upregulated by biotic agents such as infection with the fungus *Botrytis cinerea *and the bacteria *Pseudomonas syringae *pv tomato DC3000 and *Pseudomonas syringae *pv tomato avrRpm1 (Additional file 1: Figures S3C and S3D). In contrast, inoculation of leaves with *Phythophtora infestans *and *Erysiphe orontii *did not alter the expression level of ArathEULS3.

## Discussion

*In silico *analyses revealed that the recently cloned *Euonymus europaeus *lectin represents a conserved domain that is apparently widely distributed in plants and hence can be considered the prototype of what can be called the *Euonymus europaeus *lectin or EUL protein family [15]. Detailed analysis of sequences in publicly accessible databases enabled to study the distribution and the homogeneity/diversity of proteins with an EUL domain. Screening of genome and transcriptome databases indicated that proteins with EUL domains are widespread in Embryophyta (terrestrial plants). EUL sequences were found in all taxa of flowering plants (including basal Magnoliophyta, Eudicotyledons, Liliopsida, Magnoliids), in all other taxa of Spermatophyta (Coniferophyta, Cycadophyta, Ginkgophyta, Gnetophyta), and also in Filicophyta (ferns), Lycopodiophyta (e.g. *Selaginella *sp.) as well as Bryophyta (mosses) and Marchantiophyta (liverworts). Comprehensive BLAST searches of the completed genomes (and annex transcriptome) of *Chlamydomonas reinhardii*, *Chlorella *sp., *Micromonas pulsilla*, *Ostreococcus *sp., and *Volvox carteri *yielded no significant hit, suggesting that the EUL domain is absent from these Chlorophyta. Thus, it seems likely that the EUL domain was developed/acquired after the separation of the Chlorophyta and Embryophyta lineages (approximately 500 million years ago).

At present there is no evidence for the occurrence of proteins with EUL domain(s) in other eukaryotes (including green algae) or prokaryotes. Hence, one can reasonably conclude that the EUL domain is confined to the Embryophyta. It should be noted here that a few ESTs with typical EUL sequence were also found in the transcriptome of *Aedes aegypti *(an insect) whole larvae, *Wuchereria bancrofti *(a nematode) larvae, and *Xenopus laevis *whole embryos (for a complete list see Additional file 3: Table S1). However, all evidence suggests that these sequences represent contaminants arising from plant material in the respective organisms. First, all non-plant sequences are virtually identical at the nucleotide level to sequences found in Poaceae species (as is illustrated by an alignment of the sequence found in *Aedes aegypti *and an EST from the grass *Agrostis stolonifera *(Additional file 1: Figure S4). Second, the genomes of *Aedes aegypti *and *Xenopus laevis *contain no sequences that match the ESTs. Third, all non plant sequences were found in EST libraries made from complete organisms and hence can readily be contaminated with foreign cDNAs. Fourth, the apparent absence of genes encoding EUL domains from all sequenced eukaryotes other than plants is difficult to reconcile with the expression of EUL proteins in three different animal species (unless one assumes that *Aedes aegypti*, *Xenopus laevis *and *Wuchereria bancrofti *acquired in a very recent past an EUL gene from a grass species by lateral transfer). The best guess is that the larvae used for the construction of the respective EST libraries were (indeliberately) contaminated by wind carried grass pollen grains that upon RNA extraction contributed to the EST library. Accordingly, all evidence suggests that the EUL domain was developed in plants rather than acquired by either vertical or horizontal inheritance from a prokaryotic ancestor. However, it can not be precluded that other yet unidentified organisms have developed in parallel the same protein domain.

**Figure 4:**
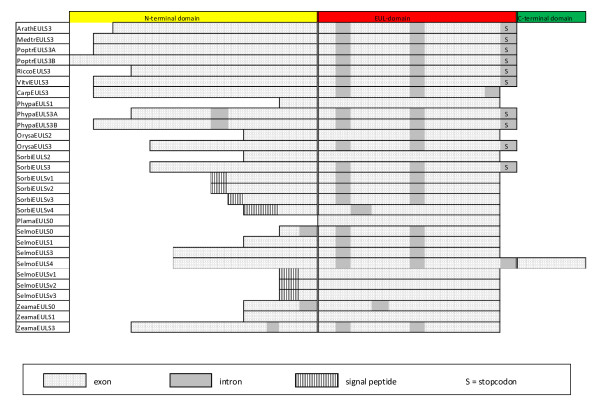
**Schematic representation of the exon/intron structure of genomic sequences containing one EUL domain**. Introns are shaded grey. Exon/intron and domain length are not drawn to scale.

**Figure 5:**
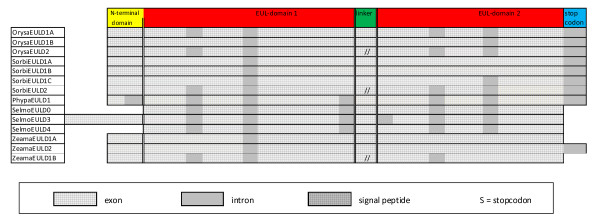
**Schematic representation of the exon/intron structure of genomic sequences containing two EUL domains**. Introns are shaded grey. Exon/intron and domain length are not drawn to scale.

A comparative analysis of the genomic and cDNA sequences revealed that most EUL sequences contain introns (Figures 4 and 5). For instance, *ArathEULS3 *contains three introns, one of which is located within the stop codon. The first exon comprises the N-terminal domain plus approximately the first 40 residues of the EUL domain whereas the rest of the EUL domain is divided over the second and third exon. A very similar exon/intron structure was also found in the genes expressing other *EULS3 *proteins though the length of the second intron can be much longer, as is the case in *PoptrEULS3A *and *PoptrEULS3B *where the second intron contains 1,397 and 3,480 nucleotides, respectively. We conclude that the position of the first and second intron in the EUL domain is conserved in all *EULS3 *genes of dicots. The third (first-order) intron is invariably located in the stop codon (Figure 4).

The EULS3 genes from monocots such as *O. sativa *and *S. bicolor *and from lower plants such as *S. moellendorfii *have exactly the same intron/exon structure as the S3-type EUL genes from dicot plant species. The *ZeamaEULS3 *gene also contains introns at the same positions as in the S3-type genes of dicots, but contains an additional intron sequence in the N-terminal domain. This is also the case for both S3-type genes of *P. patens *which contain three introns in their coding sequence and one additional intron within the stop codon. The second and third intron are located at the same position as the two introns in other S3-type genes whereas the first intron is positioned in the long N-terminal domain. An intron positioned in the N-terminal domain was also found in the S0-type gene of *S. moellendorfii*. No introns were detected in the *SorbiEULS2 *gene, while its rice ortholog contains one intron in the EUL domain (Figure 4).

Some two-domain proteins (*OrysaEULD1A*, *OrysaEULD2 *and *SorbiEULD2*) have an intron/exon structure reminiscent to that of the *EULS3 *gene (four introns in the open reading frame and one in the stop codon). However in most proteins (*OrysaEULD1B, SorbiEULD1A-C, ZeamaEULD1A-B, ZeamaEULD2) *some of these introns are apparently missing. Interestingly, all introns in the open reading frame are positioned within an EUL domain, and all expressed *EUL *genes contain a nearly identical exon sequence corresponding to the C-terminal part (comprising 76 or 77 amino acid residues) of the respective proteins. In the two-domain proteins of the lower plants *S. moellendorfii *and *P. patens *introns occur also in the N-terminal domain and/or between the linker and an EUL domain (Figure 5).

The genes encoding the vacuolar SorbiEULs have one or two introns, positioned in the EUL domain. No introns could be found in the genes encoding the presumed vacuolar EUL forms of *S. moellendorfii *(Figure 4).

Though only 12 (nearly) completed genomes have been screened, several important conclusions can be drawn with respect to the composition of EUL genes/proteins in plants. First, the genomes of dicots contain only one or two genes (or three as in the case of *Glycine max*) encoding a single-domain S3 type EUL. Second, the genome of monocots comprises a complex family of genes which encode proteins with either one or two in tandem arrayed EUL domains. Only one of these genes is a genuine ortholog of the single-domain S3-type EUL genes in dicots. The single domain S3-type EULs are present in dicots, monocots and mosses, and are markedly conserved among these divergent classes. Moreover, they are encoded by genes with a strictly conserved intron/exon structure. Therefore the S3-type EUL can be considered a universal EUL. Third, (some) lower plants such as *Selaginella *have a more complex set of EULs. Furthermore, the spikemoss *Selaginella *and the liverwort *Marchantia *express some EUL types that could not be identified in higher plants, such as EULs with an additional C-terminal domain. From these observations it is hypothesized that the EULs of seed plants evolved from the EULs of lower plants. Additionally, in some EUL genes of lower plants introns are positioned in the N-terminal domain or in the linker, which is in strong contrast to genes encoding EULs from higher plants. Nonetheless, most genes encoding EULs do typically have an intron sequence in their stop codon. Next to cytoplasmic EULs which occur in all investigated plant species, vacuolar EULs were detected only in some monocots and in *Selaginella*.

**Figure 6:**
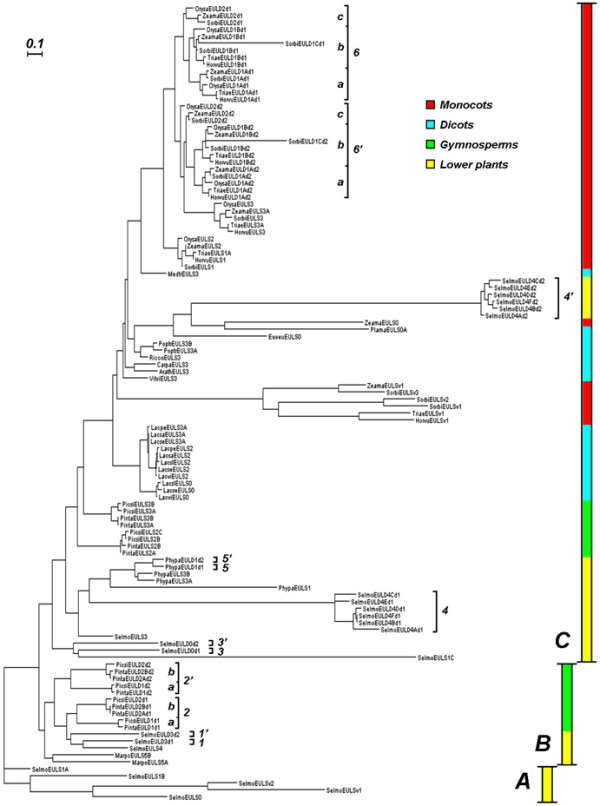
**Phylogenetic tree of proteins containing an EUL domain**. Maximum Likelihood tree depicting evolutionary relationships among EUL domains from proteins from a wide variety of plant taxa. Scale bar indicates corrected amino acid distance. Partition A represents the root (see discussion). Pairs of duplicated genes are numbered in the n, n' format, and lettered where applicable to distinguish symmetric subbranches of evolution after the duplication event. Lectin abbreviations and accession numbers or loci can be found in Additional file 3: Table S2. All sequences used for the construction of the dendrogram are listed in Additional file 1: Figure S5.

In an attempt to unravel the evolutionary relationships among sequences with EUL domains phylogenetic analyses have been performed. From the alignment of the sequences of EUL domains from different plant species it can be deduced that certain amino acids (Q_37_XW_38_XXD_41_XXXS_46_, L_60_XN_62_K_63_, H_71_, L_81_, D_90_, W_95_, D_100_, G_102_, R_109_, W_141_, N_146_Q_147_XW_149_) in this EUL domain are highly conserved (Additional file 1: Figure S2). The phylogenetic tree (Figure 6) clearly shows several clusters and some striking symmetries. Strikingly, all monocot sequences are grouped in one large cluster except for EULS0 cytoplasmic forms and the vacuolar forms retrieved from a few monocots. From the tree, a number of conclusions can be drawn with regard to the origin and evolution of the EUL domain and proteins possessing one or more EUL domains.

An outgroup for rooting the tree was designated based on the following intuitive criteria: (1) the EUL domain arose only once in the course of plant evolution, whereupon it could expand either by fusion with other segments or by gene duplication resulting in more complex EUL proteins; (2) the taxonomically most ancestral operational taxonomic units should make up the root of the tree. On these grounds SelmoEULS0, which is the oldest and simplest EUL, indeed, could be the sole outgroup member, but upon rooting with this single operational taxonomic unit it immediately became clear that the next most simple EULs (types S1 and Sv) of this species could likewise be assigned to the root partition (marked A) of the tree.

The phylogenetic phenomena thus apparent from the tree generally repeat themselves for every major taxonomic division descending sequentially from a common EUL ancestor arisen within a member of the Lycopodiophyta (*Selaginella *in this case), which are the most primitive vascular plants. Splitting off from the root of the tree there are two derived sister clusters: a smaller one (Figure 6, partition B) with lower plants and gymnosperms only, and a large one (Figure 6, partition C) with the latter plus the flowering plants (dicots and monocots). The occurrence of EUL proteins from the most primitive organisms in all major partitions of the tree, and from gymnosperms (in evolutionary terms intermediary between lower and flowering plants) in every one but the root partition clearly illustrates that EUL evolution in the first instance parallels phylogenesis as we know it today. On top of this primary pattern there are two secondary and a tertiary pattern that deserve attention while describing the clusters mentioned above: extension of single EULs, gene duplication, and reduction. Both superclusters B and C show that single EUL extension and gene duplication may have occurred at the same time, as exemplified by the appearance of EULS domains of higher order (EULS2 and above) as well as EULD domains within the more primitive taxa (e.g. the lower plants of partition B). However, the picture becomes more complicated as we consider partition C, especially when starting from its gymnosperm cluster. The latter, which according to the tree gave rise to the higher plant EUL proteins, has EULS domains of higher order; likewise, the lower plant EUL proteins above this cluster (group 4') have EULD domains of higher order. One would therefore expect the monocots and dicots, being close relatives within this partition, to express EUL proteins of higher order, yet they are mixed lower/higher. This may result from two different and not mutually exclusive factors: reduction of higher-order EULs due to deletion of unnecessary parts inherited from certain ancestors on the one hand, and on the other hand the persistence of lower-order EULs in several ancestors from which no sequences are available as yet. In any case among the higher plants there is evidence of both single EUL extension from zero (dicots and monocots) and gene duplication (monocots only); they even seem to happen independently along various lineages (e.g. a duplication event of a higher-order EUL giving rise to symmetry groups 6c and 6'c, accompanied by EULD shrinkage in symmetry groups 6a, 6'a, 6b, and 6'b independently), suggesting that flowering plants may be undergoing a renewed and accelerated cycle of EUL diversification internally, which is likely to have happened earlier among lower plants once the first EUL domain had come into being. Remnants of this early diversification can be seen in partition B, which basically displays the same patterns as partition C but to a lesser extent.

Expression profiles for the EUL from *Arabidopsis *revealed that this protein is strongly induced by some abiotic as well biotic stress factors. Similarly the expression of the so-called OSR40 proteins from rice [16,18] and some EUL proteins in maize [21] and banana [22] was shown to be stress-related. In rice the EUL proteins are specifically expressed upon salt stress and in response to ABA treatment. They have been proposed to play a role in the adaptive response of roots to a hyperosmotic environment and the response of plant tissues to salt and osmotic stresses. In maize leaves increased expression of an EUL protein was observed four days after watering of the plants was stopped [21], whereas the expression of the EUL protein in banana was much higher in a dehydration tolerant variety upon sucrose stress [22]. The expression analyses for these different EUL homologs suggest a role of these proteins associated with stress adaptation.

## Conclusion

The identification of the EUL family provides evidence for the occurrence in terrestrial plants of a highly conserved plant specific carbohydrate-binding domain. The widespread distribution of the EUL domain strikingly contrasts the more limited or even narrow distribution of most other lectin domains found in plants [2]. To our knowledge this is the first lectin family which occurs ubiquitously in plants. Previously it was shown that the EUL protein in *Euonymus *is located in the nuclear and cytoplasmic compartment [15]. Similarly most other EUL proteins identified lack a signal peptide and therefore presumably reside in the cytoplasm of the plant cell. At present all evidence from transcriptome analyses suggests that proteins with EUL domains might be involved in stress responses in plants.

## Methods

Bioinformatics analyses

EUL homology searches with the nucleotide as well as the protein sequence were done with the different BLAST programs [23] available at the NCBI website http://www.ncbi.nlm.nih.gov/BLAST/. BLAST searches were also performed against the genomic sequences of plants for which (nearly) complete genomes are available using the following databases:

- The TIGR Castor bean database http://castorbean.tigr.org

- The *Selaginella *genomics database http://selaginella.genomics.purdue.edu/; http://genome.jgi-psf.org/Selmo1/Selmo1.home.html

- The *Physcomitrella *patens genome database

http://genome.jgi-psf.org/Phypa1_1/Phypa1_1.home.html

- The moss computational biology toolbox http://www.cosmoss.org/

- The *Populus trichocarpa *genome database

http://genome.jgi-psf.org/Poptr1_1/Poptr1_1.home.html

- The *Vitis vinifera *genome database

http://www.cns.fr/spip/Vitis-vinifera-e.html

- The TIGR Rice Genome Database http://compbio.dfci.harvard.edu/cgi-bin/tgi/gimain.pl?gudb=rice

- The TIGR *Arabidopsis *database http://compbio.dfci.harvard.edu/cgi-bin/tgi/gimain.pl?gudb=arab

- The *Arabidopsis thaliana *TAIR database http://www.arabidopsis.org

- The *Arabidopsis thaliana *MIPS database http://mips.gsf.de/proj/plant/jsf/athal/index.jsp

- The *Sorghum bicolor *genome database http://www.phytozome.net/sorghum

- The *Medicago truncatula *genome database http://medicago.org/genome/IMGAG

- The TIGR Maize database http://maize.tigr.org

- The TIGR Wheat genome database http://compbio.dfci.harvard.edu/cgi-bin/tgi/gimain.pl?gudb=wheat

- The *Glycine max *genome database http://www.phytozome.net/soybean

All retrieved sequences were analyzed individually. In the absence of complete coding sequences, contigs were reconstructed from ESTs showing overlaps of at least 200 identical nucleotides. Searches were completed on February 28, 2009. Introns in the genomic sequences were identified manually by comparison to the nucleotide sequences of corresponding ESTs or cDNAs. Signal peptides were predicted with the SignalP3 program http://www.cbs.dtu.dk/services/SignalP[24] and the intracellular sorting of the proteins was analyzed using PSORT http://www.psort.org/. An electronic analysis of the expression profile of the EUL from *Arabidopsis *was performed using the *Arabidopsis *eFP Browser http://bbc.botany.utoronto.ca/efp/cgi-bin/efpWeb.cgi[19,25].

For phylogenetic analysis, an amino acid sequence dataset was compiled with one operational taxonomic unit for every single EUL domain from any known EUL protein. Sequences were aligned using ClustalW http://www.ebi.ac.uk/clustalw/[26]. Multiple alignments were visually inspected and manually corrected where necessary by means of the BioEdit package [27]. Two types of phylogenetic analysis were carried out with the help of the PHYLIP suite [28]. First, Maximum Likelihood trees were calculated (program proml) using the following relevant parameters: search for best tree (no user tree given as input to start with), no rough analysis, global rearrangements (the latter two settings effectively request an exhaustive or non-heuristic search method that takes longer to complete but yields slightly better results), random input order of sequences (number of times to jumble set to two), Jones-Taylor-Thornton model of amino acid change (which, being based on a much larger sample size, is an improved Dayhoff PAM matrix model), discrete approximation to gamma distributed rates (this feature effectively removes the artificial assumption that all positions have the same substitution rate, as further imposed by parameter settings hereafter), coefficient of variation (CV) rates 0.6 (implying that the alpha or shape parameter of the gamma distribution is 2.78, since alpha = 1/CV^2^), with the three states in the Hidden Markov Model corresponding to a rate of change of 0.498 with probability 0.532 (representing more conserved positions), 1.472 with probability 0.442 (representing medium variable positions), and 3.190 with probability 0.027 (representing hypervariable positions). Second, the latter parameter settings - wherever applicable - were used for calculating Neighbor Joining trees (program protdist followed by neighbor) for comparative purposes in order to verify the consistency of phylogenetic inference. A third phylogenetic method was applied using the MrBayes program with mixed models [29]; where possible, model parameters were left to estimate by the program itself, and the best scoring tree out of 200,000 cycles from two runs with four Markov chains each was picked for comparison with the previous two methods. Trees were visualized by either TreeIllustrator [30] or Dendroscope [31].

## List of abbreviations

AA: amino acid; ABA: abscisic acid; EEA: *Euonymus europaeus *agglutinin; EUL: *Euonymus *lectin.

## Authors' contributions

EF carried out some of the analyses, prepared the tables and figures and the primary drafts of the manuscript and contributed to finalization of the text and journal-specific formatting. WP conceived the study, analyzed genomic sequence and EST sequence information and provided valuable editorial advice. TV and MO performed the phylogenetic analyses. EVD provided overall project leadership, supervised collection, analysis and interpretation of the data, and codeveloped interim and final drafts of the manuscript. All authors read and approved the final manuscript.

## Supplementary Material

Additional file 1 **Additional Figures S1-S5**. **Figure S1**: Sequence alignment of EUL sequences with known lectin sequences. **(A, B) **Alignment of the amino acid sequences of EEA and the individual ricin-B domains of *Ricinus communis *agglutinin (AAA33869.1). **(C) **Alignment of the amino acid sequences of the EUL protein from *Curcuma longa *(CurloEULS3) and the N-terminal sequence and two tryptic peptides of the tulip lectin TxLMI. The N-terminal sequence of TxLMI is shown in bold. **Figure S2**: Multiple sequence alignment of the amino acid sequences of the EUL domain of *Euonymus europaeus *and the S3-type EULs from different plant species. The lectin abbreviations can be found in Additional file 3: Table S2. Identical residues are indicated by asterisks and similar residues by dashes or colons. The percentage sequence identity/similarity of each protein with EEA is also shown. **Figure S3**: Expression profile of the EUL from *Arabidopsis thaliana *(At2 g39050, ArathEULS3) based on the data provided by the *Arabidopsis *eFP browser. Relative expression of ArathEULS3 in the shoots of 18 day-old seedlings subjected to different abiotic stresses **(A) **and treatments with plant hormones **(B)**. Relative expression of ArathEULS3 in leaves of 4 week-old plants after infection with the pathogens *Botrytis cinerea ***(C)**, *Pseudomonas syringae *pv. tomato DC3000 (avirulent strain) and *Pseudomonas syringae *pv. tomato avrRpm1 (virulent strain) **(D)**. **Figure S4**: Alignment of EST sequence from *Aedes aegypti *(Aedae) and a nearly identical sequence from creeping bentgrass (*Agrostis stolonifera*) (Agrst). Identical nucleotides are indicated by asterisks. **Figure S5**: Amino acid sequences of proteins containing one or two *Euonymus *lectin (EUL) domains. The EUL domains are shaded yellow and green. Signal peptides are shaded grey. Only the EUL domains were used for construction of the phylogenetic tree shown in Figure 6. The first EUL-domain and the second EUL-domain of the two-domain lectins are indicated in the tree with d1 and d2, respectively. Accession numbers can be found in the Additional file 3: Table S2. Click here for file

Additional file 2 Additional data from transcriptome analyses. Click here for file

Additional file 3 **Table S1 and S2**. Table S1: List of metazoan EST sequences encoding proteins with an EUL domain. **Table S2: **Overview of sequences encoding single (S)- and double (D)-domain EULs used to construct a phylogenetic tree (Figure 6). Sequences for all EUL proteins are shown in Additional file 1: Figure S5. v: vacuolar EUL homologs. Click here for file

## References

[B1] Van Damme EJM Peumans WJ Barre A Rougé P Plant lectins: a composite of several distinct families of structurally and evolutionary related proteins with diverse biological roles Crit Rev Plant Sci 1998 17 575 692 10.1016/S0735-2689(98)00365-7

[B2] Van Damme EJM Lannoo N Peumans WJ Plant lectins Adv Bot Res 2008 48 107 209 10.1016/S0065-2296(08)00403-5

[B3] Peumans WJ Van Damme EJM Lectins as plant defense proteins Plant Physiol 1995 109 347 352 10.1104/pp.109.2.347 7480335 PMC157596

[B4] Peumans WJ Barre A Hao Q Rougé P Van Damme EJM Higher plants developed structurally different motifs to recognize foreign glycans Trends Glycosci Glycotechnol 2000 12 83 101

[B5] Van Damme EJM Barre A Rougé P Peumans WJ Cytoplasmic/nuclear plant lectins: a new story Trends Plant Sci 2004 9 484 489 10.1016/j.tplants.2004.08.003 15465683

[B6] Van Damme EJM Lannoo N Fouquaert E Peumans WJ The identification of inducible cytoplasmic/nuclear carbohydrate-binding proteins urges to develop novel concepts about the role of plant lectins Glycoconjugate J 2004 20 449 460 10.1023/B:GLYC.0000038291.67527.a5 15316278

[B7] Van Damme EJM Fouquaert E Lannoo N Vandenborre G Schouppe D Peumans WJ Wu AM Novel concepts about the role of lectins in the plant cell The Molecular Immunology of Complex Carbohydrates. New York, USA 2010 in press

[B8] Van Damme EJM Rougé P Peumans WJ Kamerling JP, Boons GJ, Lee YC, Suzuki A, Taniguchi N, Voragen AJG Carbohydrate-protein interactions: Plant lectins Comprehensive Glycoscience - From Chemistry to Systems Biology 2007 3 New York: Elsevier 563 599 full_text

[B9] Lannoo N Van Damme EJM Nucleocytoplasmic plant lectins Biochim Biophys Acta 2009 in press 19647040 10.1016/j.bbagen.2009.07.021

[B10] Lannoo N Peumans WJ Van Pamel E Alvarez R Xiong TC Hause G Mazars C Van Damme EJM Localization and *in vitro *binding studies suggest that the cytoplasmic/nuclear tobacco lectin can interact *in situ *with high-mannose and complex N-glycans FEBS Lett 2006 580 6329 6337 10.1016/j.febslet.2006.10.044 17084390

[B11] Kilpatrick DC Animal lectins: a historical introduction and overview Biochim Biophys Acta 2002 1572 187 197 12223269 10.1016/s0304-4165(02)00308-2

[B12] Sharon N Lis H History of lectins: from hemagglutinins to biological recognition molecules Glycobiology 2004 14 53R 62R 10.1093/glycob/cwh122 15229195

[B13] Haudek KC Spronk KJ Voss PG Patterson RJ Wang JL Arnoys EJ Dynamics of galectin-3 in the nucleus and cytoplasm Biochim Biophys Acta 2009 in press 19616076 10.1016/j.bbagen.2009.07.005 PMC2815258

[B14] Varki A Etzler ME Cummings RD Esko JD Varki A, Cummings RD, Esko JD, Freeze HH, Stanley P, Bertozzi CR, Hart GW, Etzler ME Discovery and classification of glycan-binding proteins Essentials of Glycobiology 2008 2 New York: Cold Spring Harbor 375 386

[B15] Fouquaert E Peumans WJ Smith DF Proost P Savvides SN Van Damme EJM The "old" *Euonymus europaeus *agglutinin represents a novel family of ubiquitous plant proteins Plant Physiol 2008 147 1316 1324 10.1104/pp.108.116764 18451263 PMC2442556

[B16] Moons A Gielen J Vandekerckhove J Straeten D Van der Gheysen G Van Montagu M An abscisic-acid- and salt-stress-responsive rice cDNA from a novel plant gene family Planta 1997 202 443 454 10.1007/s004250050148 9265787

[B17] Oda Y Minami K Isolation and characterization of a lectin from tulip bulbs, *Tulipa gesneriana* Eur J Biochem 1986 159 239 245 10.1111/j.1432-1033.1986.tb09859.x 3758061

[B18] Moons A Bauw G Prinsen E Van Montagu M Straeten D Van der Molecular and physiological responses to abscisic acid and salts in roots of salt-sensitive and salt-tolerant Indica rice varieties Plant Physiol 1995 107 177 186 10.1104/pp.107.1.177 7870812 PMC161181

[B19] Winter D Vinegar B Nahal H Ammar R Wilson GV Provart NJ An "electronic fluorescent pictograph" browser for exploring and analyzing large-scale biological data sets PLoS ONE 2007 2 e718 10.1371/journal.pone.0000718 17684564 PMC1934936

[B20] Leonhardt N Kwak JM Robert N Waner D Leonhardt G Schroeder JL Microarray expression analyses of *Arabidopsis *guard cells and isolation of a recessive abscisic acid hypersensitive protein phosphatase 2C mutant Plant Cell 2004 16 596 615 10.1105/tpc.019000 14973164 PMC385275

[B21] Riccardi F Gazeau P Jacquemot MP Vincent D Zivy M Deciphering genetic variations of proteome responses to water deficit in maize leaves Plant Physiol Bioch 2004 42 1003 1011 10.1016/j.plaphy.2004.09.009 15707837

[B22] Carpentier SC Witters E Laukens K Van Onckelen H Swennen R Panis B Banana (*Musa *spp.) as a model to study the meristem proteome: acclimation to osmotic stress Proteomics 2007 7 92 105 10.1002/pmic.200600533 17149779

[B23] Altschul SF Madden TL Schäffer AA Zhang J Zhang Z Miller W Lipman DJ Gapped BLAST and PSI-BLAST: a new generation of protein database search programs Nucleic Acids Res 1997 25 3389 3402 10.1093/nar/25.17.3389 9254694 PMC146917

[B24] Nielsen H Engelbrecht J Brunak S von Heijne G Identification of prokaryotic and eukaryotic signal peptides and prediction of their cleavage sites Protein Eng 1997 10 1 6 10.1093/protein/10.1.1 9051728

[B25] Schmid M Davison TS Henz SR Pape UJ Demar M Vingron M Schölkopf B Weigel D Lohmann JU A gene expression map of *Arabidopsis thaliana *development Nat Genet 2005 37 501 506 10.1038/ng1543 15806101

[B26] Thompson JD Higgins DG Gibson TJ CLUSTAL W: improving the sensitivity of progressive multiple sequence alignment through sequence weighting, position-specific gap penalties and weight matrix choice Nucleic Acids Res 1994 22 4673 4680 10.1093/nar/22.22.4673 7984417 PMC308517

[B27] Hall TA BioEdit: a user-friendly biological sequence alignment editor and analysis program for Windows 95/98/NT Nucleic Acids Symposium Series 1999 41 95 98

[B28] Felsenstein J PHYLIP - Phylogeny Inference Package (Version 3.2) Cladistics 1989 5 164 166

[B29] Ronquist F Huelsenbeck JP MRBAYES 3: Bayesian phylogenetic inference under mixed models Bioinformatics 2003 19 1572 1574 10.1093/bioinformatics/btg180 12912839

[B30] Trooskens G De Beule D Decouttere F Van Criekinge W Phylogenetic trees: visualizing, customizing and detecting incongruence Bioinformatics 2005 21 3801 3802 10.1093/bioinformatics/bti590 16030069

[B31] Huson DH Richter DC Rausch C Dezulian T Franz M Rupp R Dendroscope: an interactive viewer for large phylogenetic trees BMC Bioinformatics 2007 8 460 10.1186/1471-2105-8-460 18034891 PMC2216043

