# Supplementary material for: Proteins with an Euonymus lectin-like domain are ubiquitous in Embryophyta
Source: BMC Plant Biol. 2009 Nov 23;9:136. doi: 10.1186/1471-2229-9-136 (PMC2788552; doi:10.1186/1471-2229-9-136)
Supplement: Additional file 1 — Additional Figures S1-S5. Figure S1: Sequence alignment of EUL sequences with known lectin sequences. (A, B) Alignment of the amino acid sequences of EEA and the individual ricin-B domains of Ricinus communis agglutinin (AAA33869.1). (C) Alignment of the amino acid sequences of the EUL protein from Curcuma longa (CurloEULS3) and the N-terminal sequence and two tryptic peptides of the tulip lectin TxLMI. The N-terminal sequence of TxLMI is shown in bold. Figure S2: Multiple sequence alignment of the amino acid sequences of the EUL domain of Euonymus europaeus and the S3-type EULs from different plant species. The lectin abbreviations can be found in Additional file 3: Table S2. Identical residues are indicated by asterisks and similar residues by dashes or colons. The percentage sequence identity/similarity of each protein with EEA is also shown. Figure S3: Expression profile of the EUL from Arabidopsis thaliana (At2 g39050, ArathEULS3) based on the data provided by the Arabidopsis eFP browser. Relative expression of ArathEULS3 in the shoots of 18 day-old seedlings subjected to different abiotic stresses (A) and treatments with plant hormones (B). Relative expression of ArathEULS3 in leaves of 4 week-old plants after infection with the pathogens Botrytis cinerea (C), Pseudomonas syringae pv. tomato DC3000 (avirulent strain) and Pseudomonas syringae pv. tomato avrRpm1 (virulent strain) (D). Figure S4: Alignment of EST sequence from Aedes aegypti (Aedae) and a nearly identical sequence from creeping bentgrass (Agrostis stolonifera) (Agrst). Identical nucleotides are indicated by asterisks. Figure S5: Amino acid sequences of proteins containing one or two Euonymus lectin (EUL) domains. The EUL domains are shaded yellow and green. Signal peptides are shaded grey. Only the EUL domains were used for construction of the phylogenetic tree shown in Figure 6. The first EUL-domain and the second EUL-domain of the two-domain lectins are indicated in the tree with d1 and d2, resp [file 1471-2229-9-136-S1.DOC]

**Additional file 1**

**Figure S1**: **(A, B)** Alignment of the amino acid sequences of EEA and the individual ricin-B domains of *Ricinus communis* agglutinin (AAA33869.1). **(C)** Alignment of the amino acid sequences of the EUL protein from *Curcuma longa* (CurloEULS3) and the N-terminal sequence and two tryptic peptides of the tulip lectin TxLMI. The N-terminal sequence of TxLMI is shown in bold.

**A. Alignment of EEA and the N-terminal ricin-B domain of AAA33869.1 (covering residues 309-440) (18% sequence identity; 48% sequence similarity)**

EEA --PTYRVYCRAAPNYNMTVGKGVAFLAPIDETNELQYWYKDDTYSYIKDEAGLPAFSLVN 58

RicinBn PEPIVRIVGRNGLCVDVTG-------EEFFDGNPIQLWPCKSNTDWNQLWTLRKDSTIRS 53

* *: * . ::* : : * :* * ... .: : : :: .

EEA KATGLTLKHSNHHPVPVKLVTYNPNVVDESVLWSQADDRGDGYSAIRSLTNPASHLEAAP 118

RicinBn NGKCLTISKSSPR---QQVVIYNCSTATVGATRWQIWDN-------RTIINPRSGLVLAA 103

:.. **:.:*. : ::* ** ... .. * *. *:: ** * * *.

EEA LNDWSYNGAIIMGGVWIDAYNQQWKIEPHTG 149

RicinBn TS--GNSGTKLTVQTNIYAVSQGWLPTNNTQ 132

. . .*: : . * * .* * :*

**B. Alignment of EEA and the C-terminal ricin-B domain of AAA33869.1 (covering residues 441-564) (11% sequence identity; 44% sequence similarity)**

EEA PTYRVYCRAAPNYNMTVGKGVAFLAPIDETNELQYWYKDDTYSYIKDEAGLPAFSLVNKA 60

RicinBc -PFVTTIVGLYGMCLQANSGKVWLEDCTSEKAEQQWALYADGSIRPQQ---------NRD 50

.: . . . : ...* .:* . : * * * :: *:

EEA TGLTLKHSNHHPVPVKLVTYNPNVVDESVLWSQADDRGDGYS-AIRSLTNPASHLEAAPL 119

RicinBc NCLTT-DANIKGTVVKILSCGPASSGQRWMFKNDGTILNLYNGLVLDVRRSDPSLKQIIV 109

. ** .:* : . **::: .* .: ::.: . : *. : .: .. . *: :

EEA NDWSYNGAIIMGGVWIDAYNQQWKIEPHTG 149

RicinBc HPFHGN----LNQIWLPLF----------- 124

: : * :. :*: :

**C. Alignment of CurloEULS3 with the N-terminal sequence and internal tryptic peptides of TxLMI**

CurloEULS3 GCPPGGHGGRVEEPSKLRQPTVRIFTRADENYSLSIRDGKVVLVRNDPGDQYQHWIKDMR 60

TxLMI ---------------------**MRIYTKVDESYSLAARDGKVV**---SDSGDQNQQWFR

:**:*:.**.***: ****** .*.*** *:*::

CurloEULS3 YSTKVKDQEGFPSFCLINKAPGEALKHSIGATHPVRLVPYNPDYLDESVLWTESGDTGNG 120

TxLMI ------NQDGSASFALV

:*:* .**.*:

CurloEULS3 FRCIRMVNNIGVNFDAFHGDKDHGGVRDGTPVVLWEWLKGHNQQWKIVPS 170

TxLMI

**Figure S2:** Multiple sequence alignment of the amino acid sequences of the EUL domain of *Euonymus europaeus* and the S3-type EULs from different plant species. The lectin abbreviations can be found in Additional file 3: Table S2. Identical residues are indicated by asterisks and similar residues by dashes or colons. The percentage sequence identity/similarity of each protein with EEA is also shown.

| % identity with EEA | % similarity with EEA |
| --- | --- |
| 44 | 71 |
| 40 | 75 |
| 40 | 76 |
| 42 | 75 |
| 46 | 77 |
| 40 | 74 |
| 46 | 77 |
| 39 | 70 |
| 40 | 68 |
| 39 | 70 |
| 38 | 70 |
| 41 | 70 |
| 100 | 100 |
| 34 | 71 |
| 34 | 68 |
| 37 | 66 |
| 38 | 68 |
| 38 | 68 |

ArathEULS3 AGRATVKVYSKAEPN-YNLTIR-DGKVILAPADPSDEAQHWYKDEKYSTKVKDADGHPCF 58

MedtrEULS3 ---STFKIVTKASPN-YSLTIR-RGEVVLAPSDPSDQHQHWYKDVKWSTKVKDKDGYPSF 55

PoptrEULS3A ---PSFKVYSKAEPD-FHLTIR-DGRVILSRSNPSDEFQNWFKDEKYSTRVKDSEGCPAF 55

PoptrEULS3B ---PSFKVYSKAQPE-FHLTIR-GGKVILAPSNPSDEFQNWYKDEKYSTRVKDSEGCPAF 55

RiccoEULS3 ---PTYKVYSKAEPN-FHLTIR-DGKVVLAPSDPSDEFQNWYKDERYSTRVKDEEGFPCF 55

LacpeEULS3A ---PTVRFYSKIKTN-YSLTIR-NGEVILAPTNPSDYHQHWIKDEKFSTRVKDEEGFPSF 55

VitviEULS3 ---PTVRVFCKAKPN-HSLTIL-DGKVYLAPSDKTDMLQHWIKDEKYSTSVKDEEGFPSF 55

SorbiEULS3 --QQTYRIYCKAGDDQYSLAAR-DGKVCLVRTERNDDTQHWIKDMKYSTRVKDEEGYPAI 57

ZeamaEULS3A ---QTYRIYCKAGEDQYSLASR-DGKVCLVRTDRDDDAQHWIKDMKYSTRVKDEEGYPAI 56

HorvuEULS3 ---PTYRVFCKAGEESFNLAAR-DRKVCLVRTDRDDDTQHWIKDMKYSTRVKDEEGYPAM 56

TriaeEULS3A ---PTYRILCKAGEDSFSLAAR-DGKVCLVRTDRDDDTQHWIKDMKYSTRVKDEEGYPAM 56

OrysaEULS3 ---VTHRIYCKAGEDNYSLAVR-DGKVCLVRSDRDDHTQHWVKDMKYSTRVKDEEGYPAM 56

EEA ---PTYRVYCRAAPN-YNMTVG-KGVAFLAPIDETNELQYWYKDDTYS-YIKDEAGLPAF 54

PhypaEULS3A ---LPVRLHCKADPN-FNLAAVPGQGPVMVPFSPNDDFQVWYKDVTMSTRVKDETGSSAF 56

PhypaEULS3B ----PVRIHCKADPS-YNLAVVPGQGPVMVPTDISDDYQVWYKDETISTRVTDETGASAF 55

SelmoEULS3 -LGQLVKLCCQANMD-FSLAVR-DDGVVLVPSNHHDDSQQWYKDMSWSTRVRDEKGFPAF 57

PintaEULS3A --GQVVRVFNKANPD-FWLAIR-DGSTVLVHANPNDESQQWVMDETYSTKVKDEASSPSF 56

PicsiEULS3A --GQVVRVLTKANPD-FSLAIR-NGSAVLVHENANDKHQQWVMDETYSTKVKDEASCPSF 56

:. : . . :: : . : * * * * : * . ..:

ArathEULS3 ALVNKATGEAMKHSVGATHPVHLIRYVPDKLDESVLWTESKDFGDGYRTIRMVNNTRLNV 118

MedtrEULS3 ALVNKVTGEAIKHSIGASHPVRLIRYNPDYLDQSVLWTESKDQGSGYRAVRMVNNIQLNM 115

PoptrEULS3A ALVNKATGQAIKHSIGEANPVQLIPYNPDVLDQSILWTQSKDLGDGFRAVRMVNNTHLNV 115

PoptrEULS3B ALVNKATGQAMKHSIGEAHPVQLIPYNPDVLDESILWTESKDLGDGFRAVRMVNNTHLNV 115

RiccoEULS3 ALVNKASGQAMKHSIGGTHPVQLIPYNPNVLDESILWTESKDLGDGYRAVRMVNNIHLNV 115

LacpeEULS3A ALVNKATGQALKHATGAAKPVQLTEYNPDKLDESVLWTQSKDLGDGFHAVRMVNKIKLNV 115

VitviEULS3 ALVNKATGQAMKHSIGASHPVQLIPYNPDVLDESVLWTESKDLGDNFRSIRMINNIHLNV 115

SorbiEULS3 VLVNKATGEALKHSLGQSHPVPLTRYDPDKLDESVLWTESRDVGDGFRCIRMVNNIYLNF 117

ZeamaEULS3A VLVNKATGEALKHSLGQSHPVLLTRHNPDSLDESVLWTESRDVGDGFRCIRMVNNIYLNF 116

HorvuEULS3 ALVNKASGEALKHSLGQSHPVLLTRYNPDTLDESVLWTESRDVGAGYRCIRMVNNIYLNF 116

TriaeEULS3A ALVNKASGEALKHSLGQSHPVRLTRYNPDTLDESVLWTESRDVGAGYRCIRMVNNIYLNF 116

OrysaEULS3 ALVNKATGDALKHSIGQSHPVRLVRYNPEYMDESVLWTESRDVGSGFRCIRMVNNIYLNF 116

EEA SLVNKATGLTLKHSNHHPVPVKLVTYNPNVVDESVLWSQADDRGDGYSAIRSLTNPASHL 114

PhypaEULS3A SLINKATGQALRHAPEDLAQCLLADYDSNALDQTVLWTMSEDMGQGYCCIRLASQITRNL 116

PhypaEULS3B SLINKATGQALRHAPEDLKQCLLTQYEPNGLDDTIWWTMSEDMGQGYHCIRLATDITRNM 115

SelmoEULS3 ALINKATRKALKHATEELQQVLLVDYNPRVVDESVLWTVSEDMGSGFRTIRMASNIKLNL 117

PintaEULS3A SLVNKATGQALKHGLGETQPVLLTQYDPNNFDESILWTTSGDMGQGFRTIRLVNNIHLNL 116

PicsiEULS3A SLVNKATGQVLKHGLGETQPVLLTEYQLNTFDESILWTMSGDMGQGYRTIRLVNNIHLNL 116

*:**.: .::*. * : .*::: *: : * * .: :* .. :.

ArathEULS3 DAYHGDSKSGGVRDGTTIVLWDWNKGDNQLWKIFPF-- 154

MedtrEULS3 DAFHGDKNSGGVHDGTTVVLWDWNKGDNQQWKILP--- 150

PoptrEULS3A DAFHGDKKSGGVHDGTTIVLWKWNKGDNQRWKIIPAQY 153

PoptrEULS3B DAFHGDKKSGGVHDGTSIVLWKWNKGDNQRWKIIPTRY 153

RiccoEULS3 DAFHGDKKSGGVHNGTTIVLWKWNKGDNQRWRITPH-- 151

LacpeEULS3A DASIGDT---GIHDGTKILCWEWTKGDNQRWTTAPF-- 148

VitviEULS3 DALHGDRSHGSVHDCTTIVLNKWKKGDNQLWKISLY-- 151

SorbiEULS3 DALHGDKDHGGVHDGTTLVLWEWCEGDNQRWKIVPW-- 153

ZeamaEULS3A DALHGDKDHGGVRDGTALVLWEWCEGDNQRWKIVPW-- 152

HorvuEULS3 DALHGDKDHGGVRDGTTLILWEWTEGDNQRWKIVAW-- 152

TriaeEULS3A DALHGDKDHGGVRDGTTLILWEWTEGDNQRWKIVAW-- 152

OrysaEULS3 DALHGDKDHGGVRDGTTLVLWEWCEGDNQRWKIVPW-- 152

EEA EAAPLNDWS---YNGAIIMGGVWIDAYNQQWKIEPHTG 149

PhypaEULS3A DVLRGDKKSGGVKEGSPVITFAWKKQDNQIWKMITA-- 152

PhypaEULS3B DVLRGDKKSGGVKEGSPVITFAWKKQDNQIWKMTPA-- 151

SelmoEULS3 DAFRGDKRSGGVKDGTPAVLYRWKKQENQLWKIIPL-- 153

PintaEULS3A DAFHGDKKSGGIKDGNPVVLWSWKKGDNQLWKIVPF-- 152

PicsiEULS3A DAFHGDKKSGGIKDGDPVVLWSWKKGDNQLWKIVPY-- 152

:. : : : * . ** *

**Figure S3:** Expression profile of the EUL from *Arabidopsis thaliana* (At2g39050, ArathEULS3) based on the data provided by the *Arabidopsis* eFP browser. Relative expression of ArathEULS3 in the shoots of 18 day-old seedlings subjected to different abiotic stresses **(A)** and treatments with plant hormones **(B)**. Relative expression of ArathEULS3 in leaves of 4 week-old plants after infection with the pathogens *Botrytis cinerea* **(C)**, *Pseudomonas syringae* pv. tomato DC3000 (avirulent strain) and *Pseudomonas syringae* pv. tomato avrRpm1 (virulent strain) **(D)**.


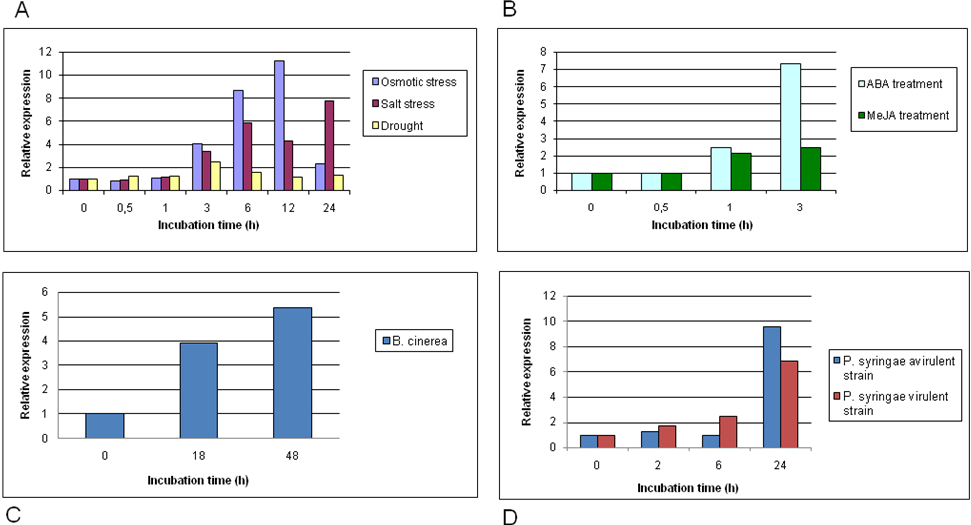


**Figure S4**: Alignment of EST sequence from *Aedes aegypti* (Aedae) and a nearly identical sequence from creeping bentgrass (*Agrostis stolonifera*) (Agrst). Identical nucleotides are indicated by asterisks.

Aedae ---GGAATTCCCGGGATCACCAGCATCAAGGACGAGGAGGGGTACCCTGCCTTCGCCCTG

Agrst AAGGACATGAGGTGGAGCACCAGCATCAAGGACGAGGAGGGATACCCTCCCTTCGCCCTG

* ** *** ************************ ****** ***********

Aedae GTGAACAAGGCCACCGGGGAGGCCATCAAGCACTCCCTTGGCCAGTCCCACGCTGTTCGG

Agrst GTGAACAAGGCCACAGGGGAGGCCGTCAAGCACTCCCTTGGCCAGTCCCACCCTGTTCGG

************** ********* ************************** ********

Aedae CTTGTGCCGTACAACCCAGACTACCTGGACGAGTCGGTGCTGTGGACGGAGAGCAGGGAC

Agrst CTTGTGCCGTACAACCCTGACTACCTGGACGAGTCGGTGCTGTGGACGGAGAGCCGGGAC

***************** ************************************ *****

Aedae GTGGGCAACGGCTTCCGCTGCGTGCGCATGGTGAACAACATCTACCTCAACTTCGACGCC

Agrst GTGGGCAACGGCTTCCGCTGCGTGCGCATGGTGAACAACATCTACCTGAACTTCGACGCC

*********************************************** ************

Aedae CTCAACGGCGACAAGTACCACGGCGGCGTCCGCGACGGCACCGAGGTCGTGCTCTGGAAG

Agrst CTCAACGGCGACAAGTACCACGGGGGCGTCCGCGACGGCACCGAGGTCGTGCTTTGGAAG

*********************** ***************************** ******

Aedae TGGTGCGAGGGCGACAACCAGCGCTGGAAGATCCAGCCATACTACTGA

Agrst TGGTGGGAGGGGGACAACCAGCGCTGGAAGATCCAGCCATACTACTGA

***** ***** ************************************

**Figure S5**: Amino acid sequences of proteins containing one or two *Euonymus* lectin (EUL) domains. The EUL domains are shaded yellow and green. Signal peptides are shaded grey. Only the EUL domains were used for construction of the phylogenetic tree shown in Figure 6. The first EUL-domain and the second EUL-domain of the two-domain lectins are indicated in the tree with d1 and d2, respectively. Accession numbers can be found in the Additional file 3: Table S2.

**One-domain lectins**

***Arabidopsis thaliana***

>ArathEULS3

M E H H H Q H H R H H Q R D D G E D D R Q S F G V P P P H V D A P P Q P H G L Y Q S Q P H F D P Y A P T P Q A P A P Y R S E T Q F E P H A P P P Y R S E P Y F E T P A P P P S F G H V S H V G H Q S P N E S Y P P E H H R Y G G Y Q Q P S N S L L E S H G D H S G V T H V A H H S S N Q P Q S S S G V Y H K P D E N R L P D N L A G L A G R A T V K V Y S K A E P N Y N L T I R D G K V I L A P A D P S D E A Q H W Y K D E K Y S T K V K D A D G H P C F A L V N K A T G E A M K H S V G A T H P V H L I R Y V P D K L D E S V L W T E S K D F G D G Y R T I R M V N N T R L N V D A Y H G D S K S G G V R D G T T I V L W D W N K G D N Q L W K I F P F

***Euonymus europaeus***

>EuoeuEULS0

M A S T I I A T G P T Y R V Y C R A A P N Y N M T V G K G V A F L A P I D E T N E L Q Y W Y K D D T Y S Y I K D E A G L P A F S L V N K A T G L T L K H S N H H P V P V K L V T Y N P N V V D E S V L W S Q A D D R G D G Y S A I R S L T N P A S H L E A A P L N D W S Y N G A I I M G G V W I D A Y N Q Q W K I E P H T G

***Carica papaya***

>CarpaEULS3

**M** E F P F G H H H H H H H R R N D E E D D R E R P H Y P P P S A A E F S D P P P P P P P S Y Y Q H N E F E P P T R V T H T H H S A G F N Q Q D P D S F N Y Q P R P P T Q V T H L H H S G D Y P P E S G Y H N Y P P E P E **M** T H G Y R H D Q R P G Y H N Y A P A P E T E V I Q F S H Q G N P D S Y R P E A E T H D P F R P H L P S F L H Q H T H Q S S G D G L S S R R T V R V F C K A N P D Y S L A I R D G K V I L V R S D P R D E Y Q H W F K D E K Y S T R V K D E E G L P S F A L V N K A T G E A **M** K H S I G D T H P V Q L R P Y K P D D F D E S V L W S Q S K D F G D D F R T I R **M** V N N T R L N V D A F H G D K K S G G V R E G T I I V L W K W N K G D N Q L W K I R P F D A

***Hordeum vulgare***

>HorvuEULS1

M D Y Y R E S Y G S Y G M A T P G Y A P P V P Y G M S Q V N V D G N C G G R P M P P Q P T V K I Y C R A N P N Y A M S V R N G K V V L A P A N P K D D Y Q H W I K D M R W S T S I K D E E G Y P A F A I V N K A T G Q A I K H S L G Q S H P V R L V P Y N P D F L D E S V L W T E S R D V G N G F R C V R M V N N I Y L N F D A L N G D K Y H G G V R D G T E V V L W K W C E G D N Q R W K I Q P Y Y

>HorvuEULS3

M E F P H R H G H G H G R R D D D D D D R R P P A P Y G R Q E P D P Y G A P P P C Y G R R P D D V A Y G A P P P A Y G G G R E D D Y G G R A P A Y G G G R E D D Y G G R A P A Y G G G R E D D Y G G R A P A Y G G G R E D D Y G R H A P A P A G Y G G G D Y G R H A P A P A Y G G G R D E G Y G A P A H G N V V H V S H E S G D E R P Q Y G G Y G N E T R P H H G G G G G M A P P A T R Q P T Y R V F C K A G E E S F N L A A R D R K V C L V R T D R D D D T Q H W I K D M K Y S T R V K D E E G Y P A M A L V N K A S G E A L K H S L G Q S H P V L L T R Y N P D T L D E S V L W T E S R D V G A G Y R C I R M V N N I Y L N F D A L H G D K D H G G V R D G T T L I L W E W T E G D N Q R W K I V A W

>HorvuEULSv1

M A A A A G L V W V T L L M C V A L G A G Q L D R A S I Q K A L V A A F F P E G S L P P P V R I Y C R E D A A L N V G I D G N N N V V L V N A D C S D L S Q K W F P V Y I R S S L D N G E I E R K P F F L M N A Q T S Q V I T I P T W S R S T G Q K V G L S S P P T T L L L V N T W L Q E A S K Q L W T P E K P T R T D G F Y K L I V T N D E D A S L N G L L G G V K V G T E V G I Y Y S S A N S D N A V W K L T S S I T N C Y P

***Lactuca perennis***

>LacpeEULS2

M D H H H N N A P S H H Q T P Q P A V A V H H A P A Q T H H Q S S P Q P A A A V H H V S H Q T P K Y T D N K P T V R F Y S K I K T D Y S L T I R N G E A V L A P T N P S D P H Q H W I K E E K L S T K V K D E E G F P S F A L I N K A T G Q A L K H A T G S A K P V Q L T E Y N P D R V D E S V L W T Q S K D L G D G F H A V R M V N K I K L N I D A S I G D T G I H D G T K I L C W E W T K G D N Q R W T T A P F

>LacpeEULS3A

M D P R H H H A H Q P T P P P P Q P A H H I P A H H Q P S P P L P Q H H A P T H H Q P S P P P P Q H H A P T H H Q Q S P P P P Q P A H H A S S H H Q P S P P P P Q P A A V V H H V S H Q S P K Y T D N K P T V R F Y S K I K T N Y S L T I R N G E V I L A P T N P S D Y H Q H W I K D E K F S T R V K D E E G F P S F A L V N K A T G Q A L K H A T G A A K P V Q L T E Y N P D K L D E S V L W T Q S K D L G D G F H A V R M V N K I K L N V D A S I G D T G I H D G T K I L C W E W T K G D N Q R W T T A P F

***Lactuca saligna***

>LacslEULS0

M D Q T P K Y T D D K P T V R F Y S K I K T D Y S L T I R D G A P V I T P T N S S D L H Q H W I K D E K F S T R V K D E E G F P S F A L V N K A T G Q A L Q Q P T D P A H K P V Q L T E F N P N T L D L S V L W T E S K D L G D S F H A V R M V D N I K L N I D A S I G D T G I H D G T E I P L W E W N K N D N Q R W K T E P F

>LacslEULS2

M D H H H N K A P S H H Q S T P Q P A V A V H H A P A Q T H H Q S S S Q P A A V Q H V S H Q S P K Y T D N K P T V R F Y S K I K T D Y S L T I R N G E P V L A P T N P S D P H Q H W I K E E K L S T K V K D E E G F P S F A L I N K A T G Q A L K H A T G A A K P V Q L T E Y N P D R V D E S V L W T Q S K D L G D G F H S V R M V N K I K L N I D A S I G D T G I H D G T K I L C W E W T K G D N Q R W T T A P I

***Lactuca sativa***

>LacsaEULS2

M D H H H N N A P S H H Q S T P Q P A V A V H H A P A Q T H H Q S S S Q P A A V Q H V S H Q S P K Y T D N K P T V R F Y S K I K T D Y S L T I R N G E A V L A P T N P S D P H Q H W I K E E K L S T K V K D E E G F P S F A L I N K A T G Q A L K H A T G A A K P V Q L T E Y N P D R V D E S V L W T Q S K D L G D G F H S V R M V N K I K L N I D A S I G D T G I H D G T K I L C W E W T K G D N Q R W T T A P F

>LacsaEULS3A

M D P R H H H N A Y Q P T P P P P Q P A H H L P T H H Q P S P P P P Q H H A P A H H Q P S P P P P Q P S H H V T S H H Q P S P P P P Q P A A I V H H V S H Q T T H Q T P K Y T D N K P T V R F Y S K I K T N Y S L T I R N G E V V L A P T N P S D H H Q H W I K E E K F S T R V K D E E G F P S F A L V N K A T G Q A L K H A T G A A K P V Q L T E Y N P D K L D E S V L W T Q S K D L G D G F H A V R M V N K I K L N V D A S I G D T G I H D G T K I L C W E W T K G D N Q R W T T A P F

***Lactuca serriola***

>LacseEULS0

M D Q T P K Y T D N K P T V R F Y T K I K T D Y S L T I H D G S P V I A P T N S S D L H Q H W I K D E K F S T R V K D E E G F P S F A L V N K A T G Q A L Q Q P T D P A H K P V Q L T E F N P Y T L D L S V L W T E S K D L G D G F H A V R M V D N I K L N I D A S I G D T G I H D G T E I L L W E W N K N D N Q R W K T E P F

>LacseEULS2

M D H H H N N A P S H H Q S T P Q P A V A V H H A P A Q T H H Q S S S Q P A A V Q H V S H Q S P K Y T D N K P T V R F Y S K I K T D Y S L T I R N G E A V L A P T N P S D P H Q H W I K E E K L S T K V K D E E G F P S F A L I N K A T G Q A L K H A T G A A K P V Q L T E Y N P D R V D E S V L W T Q S K D L G D G F H S V R M V N K I K L N V D A S I G D T G I H D G T K I L C W E W T K G D N Q R W T T A P F

>LacseEULS3A

M D P R H H H N A Y Q P T P P P P Q P A H H L P T H H Q P S P P P P Q H H A P S H H Q P S P P P P Q H H A P A H H Q P S P P P P Q P S H H V T S H H Q P S P P P P Q P A A I V H H V S H Q T T H Q T P K Y T D N K P T V R F Y S K I K T N Y S L T I R N G E V V L A P T N P S D H H Q H W I K E E K F S T R V K D E E G F P S F A L V N K A T G Q A L K H A T G A A K P V Q L T E Y N P D K L D E S V L W T Q S K D L G D G F H A V R M V N K I K L N V D A S I G D T G I H D G T K I L C W E W T K G D N Q R W T T A P F

***Lactuca virosa***

>LacviEULS0

M D Q T P K Y T D N K P T V R F Y S K I K T D Y S L T I R D G A P V I A P T N S S D L H Q H W I K D E K F S T R V K D E E G F P S F A L V N K A T G Q A L K Q A T D P A H N P V Q L T E F N P N T L D L S V L W T E S K D L G D G F H A V R M V D N I K L N I D A S I G D T G I H D G T E I L L W E W N K N D N Q R W K T E P F

>LacviEULS2

M D H H H N N A P S H H Q S T P Q P A V A V H H A P A Q T H H Q S S S Q P A A V Q H V S H Q T P K Y T D N K P T V R F Y S K I K T D Y S L T I R N G E A V L A P T N P S D P H Q H W I K E E K L S T K V K D E E G F P S F A L V N K A T G Q A L K H A T G A A K P V Q L T E F N P D R V D E S V L W T Q S K D L G D G F H S V R M V N K I K L N V D A S I G D T G I H D G T K I L C W E W T K G D N Q R W T T A P F

***Marchantia polymorpha***

>MarpoEULS5a

M A A E E H Y G H H H L H R H E H R S N L H M T H N N P G G Y N P A H P T G E L V S V F S E A N P N Y R L A V K P D G V V L A F N N R H D P Q Q Q W I K V D M R D K F T D Q Q G C P G F I L I N K A T G M A L K H G T E L G D P V T A E L W R P N Y L D N S I L W S Q S S D V G K G Y S T I R L V T N I T L N L D A D H G D R K H G G I K D G H R L L L H T W T K G E N Q K W K M E P V E V E P E P S P Y D S

>MarpoEULS5b

M S Y G E G E G Y F R R S N D E G Y A H Y P H H Q H G H V D P P P R R E T M E V T G E I L K I F C E A R P D F N L A V R H H E V V L V P G D D N D P N Q T W I K D E S W S T K V K D S A G F P A F A L V N R G T G K A L L H H I E A P D S P V M V E D Y E M N V L N E A V L W T A S K D V G N G Y R A I R P V S N I H L N L D A D H G S E K Y G G V K D G N K V I L F K W K D E P N Q H W H M A P V E L Q R D H H Y R P Q E Y E Y E R R P V D H H S R S H E R E Y E Q R H D H H S H S R E R E R E Y E R E H H G Q S H H S H S H P G Y Y

***Medicago truncatula***

>MedtrEULS3

M E F P F N H N T S N V T H T H H H R R D D D N N E Q H Y P P P G H N N L S S F N Q P P P P P H H Q Q P P F Y P N P S Y P P P P Q Q Q P H Q P E T Q V F H T G H V S R N D N F N N Y P Q Q P Q P H Q E T Q V F H T G H V S H E N F N S Y P Q P P P P Q S H H Q Q P S Y G A A Y P P A P P S V P D H T S A P F P N A T V H H V S H E T H N P H F P S S N V H H V N H E P T A P P L S S N R S T F K I V T K A S P N Y S L T I R R G E V V L A P S D P S D Q H Q H W Y K D V K W S T K V K D K D G Y P S F A L V N K V T G E A I K H S I G A S H P V R L I R Y N P D Y L D Q S V L W T E S K D Q G S G Y R A V R M V N N I Q L N M D A F H G D K N S G G V H D G T T V V L W D W N K G D N Q Q W K I L P

***Oryza sativa***

>OrysaEULS2

M D F Y G R R E Q Y G G Y G G Y G G G G A L A T P G Y A P A A P Y G M S Q V N I E G N G C G R T L P P Q P T V K V Y C R A N P N Y A M T A R N G A V V L A P A N P K D E Y Q H W I K D M R W S T S I K D E E G Y P A F A L V N K A T G Q A I K H S L G Q S H P V R L V P Y N P E V M D E S V L W T E S R D V G N G F R C I R M V N N I Y L N F D A F H G D K Y H G G V R D G T D I V L W K W C E G D N Q R W K I Q P Y Y

>OrysaEULS3

M E F P H R H H H H G H R G D D D D D D R R R H P A P A P A Y G H D S A P P P G P Y G Q A P P P A D P Y A R H P P S H D Y A H P P P A Y G G G G Y G N V V H V S H E V S D H Q R P T P H Y G G S E Y I S P V Q E T R P Y H G G G G A P P V T H R I Y C K A G E D N Y S L A V R D G K V C L V R S D R D D H T Q H W V K D M K Y S T R V K D E E G Y P A M A L V N K A T G D A L K H S I G Q S H P V R L V R Y N P E Y M D E S V L W T E S R D V G S G F R C I R M V N N I Y L N F D A L H G D K D H G G V R D G T T L V L W E W C E G D N Q R W K I V P W

***Plantago major***

>PlamaEULS0A

**M** A G T N G S C E S Y R L Y C K R Q G Q A G G N Y C V T A R Q G K L Y L T H P N G A D C N Q I W Y K V G N D K V G V I **M** L V H K A T G **M** V V K H S T T G V Q L Q L V K K P S G C A D K S V L W A E S A D V G S G Y R F L K T Q T D T N Y V **M** D A W T G I I N E G T I I S I Y P N C I N D S N R N A E N Q L W K L A P

***Physcomitrella patens***

>PhypaEULS3A

M N R G E G E P Y N P Y P E K E G H H H H H H H H H R H F S D E E Q P R Y G S N L E H R P P P M P V S G D A A P Y G Y G E G S Y G Q E G R R G A Y E E Q G Y R H S G G Y P P S G P R Y V G P E Y H N S P Y V P A P R H H S V V T E D E G S R R R P V L G L P V R L H C K A D P N F N L A A V P G Q G P V M V P F S P N D D F Q V W Y K D V T M S T R V K D E T G S S A F S L I N K A T G Q A L R H A P E D L A Q C L L A D Y D S N A L D Q T V L W T M S E D M G Q G Y C C I R L A S Q I T R N L D V L R G D K K S G G V K E G S P V I T F A W K K Q D N Q I W K M I T A

>PhypaEULS3B

M H R G E R E P Y N P Y P E E G Y G R G E E F P R R D H H H P H H P H H S S E A G P Q H G M N L E H R P P P P V V Y G G S A P H G Y S E G P Y G Q E R R P G P Y E E Q K P G G H E G Y T D Y R H G G Y E D R R N T S K Y P T G S Y V Q M D S L P G G Y D A G P P S G H G Y G S A E Y N T S P Y A P A P H H R A R D E D E G P R R R Q F P G H P V R I H C K A D P S Y N L A V V P G Q G P V M V P T D I S D D Y Q V W Y K D E T I S T R V T D E T G A S A F S L I N K A T G Q A L R H A P E D L K Q C L L T Q Y E P N G L D D T I W W T M S E D M G Q G Y H C I R L A T D I T R N M D V L R G D K K S G G V K E G S P V I T F A W K K Q D N Q I W K M T P A

>PhypaEULS1

M C K Q I C K V C N G I C T C H Y C K G A E T V R I H S K A N A N Y N V A V R G T V G H G T C I V M A D D T D S S Q L W L K D D K M A W T F D K K G G F S I V H K D T M K A I R V A P E E G K Q V L L A D Y D P R K L D E S L I W Y Q S S N R G R N Y H T I Y N G T S E T V L H A L R C T Q C E L M Q P C T E G N A H M K E N T L V V V M K D R T K K D D P A T V A M N Q L W K L T P S K C R F

***Picea sitchensis***

>PicsiEULS2B

M A Y Y G E Y D P C Q E R N A S G S G F Q Q Q Q L A R P P C D P C Q N A T Q E G Y G F N E Y G D R A A C N K F N A G G G C M R N S Q Q I I P Q G Q I V R V C S K A N P D Y A L A I R D G R A V M V F Y N P N D P T M Q W V K D E S W S N Q V R D Q V G H P A F A L V N K A T G Q A L R H A I A E C Q E V L L T Q Y E G A S T Y D E N V L W S E S E D M G Y G Y R T V R M A N N I G L N L D A F Q G D R R N G G I R D G T R A V L W K W N K Q D N Q L W K L S P C Y

>PicsiEULS2C

M A Y Y G E Y D S C Q G R N E R V Y E S P P N S G Y G Y H Q Q P A R P P C D P C L N A T D G G Y G F N D Y G G P A S R N K F N A T G G Y M M S G Q Q L I P Q G Q I V R V C S K A N P D Y S L A I R D G R A V M V F C N L N D P T M Q W V K D E S W S N Q V R D Q V G H P A F A L V N K A T G Q A L R H A I A E C Q E V L L T Q Y E G A S T Y D E N I L W S E S E D M G Y G Y R T V R M A N N I G L N L D A F Q G D R R N G G I R D G T R A V L W K W N K Q D N Q L W K L S P C Y

>PicsiEULS3A

M E F P Y G G Q P H H R R E D H E E E R R E H Q H H H Q G F Q P S P D F P D R P S A G E Y H P P P G T Q F P E P G Y Y P P P P Q P Q P Y S Q V E H H A H Q P F S Q G E E H H A Y Q G S G H V I H H E S R H P F Q R P E C E T P S D S F P R P S S G T S E G S F T R P G Y G P P G G S F P R P G G E P V L P R G Q V V R V L T K A N P D F S L A I R N G S A V L V H E N A N D K H Q Q W V M D E T Y S T K V K D E A S C P S F S L V N K A T G Q V L K H G L G E T Q P V L L T E Y Q L N T F D E S I L W T M S G D M G Q G Y R T I R L V N N I H L N L D A F H G D K K S G G I K D G D P V V L W S W K K G D N Q L W K I V P Y

>PicsiEULS3B

M E F P Q G R Q P H N C H Q D H E E E R R E Q Q H H H H G F Q P S P V F P R P S S A G E Y Y P P P G T Q F P E P G Y Y P P P P P P Q P Y S Q V E H H A H Q P F S Q G E E P H A H Q G F G H V I H H E S H H P F Q R P E C E T P S G S F P R P S C G S S E N S F T R P G Y G P P G G S F P R P E R E P V L P R G Q V V R V F T K A N P D F S L A I R N G S A V L V H A N A H D K H Q Q W V N D E T Y S T K V K D E A N C P S F S L V N K A T G Q A L K H G L G E T Q P V L L T E Y Q L N T F D E S I L W T M S G D M G Q G Y R T I R L V N N I H L N L D A F H G D K K S G G I K D G N P V V L W S W K K G D N Q L W K I V P Y

***Pinus taeda***

>PintaEULS2A

M A Y Y G E Y N P C Q E R S A S G Y G Y Q Q Q P A R P P C K Y G G R A A C N N L N A G G G C M M S G Q Q I I P Q G Q I V R V C S K A N P D Y A L A I R D G R A V M A F Y N P N D P T M Q W V K D E T W S N Q V R D Q V G H P A F A L V N K A T G Q A L R H A I A E C Q E V L L T Q Y E G P S T Y D E N I L W S E S E D M G Y G Y R T I R M A N N I G L N L D A F H G D R R S G G I R D G T R A V L W K W N K Q D N Q L W K L S P C Y

>PintaEULS2B

M A Y Y G E Y D S C Q E R N Q R I Y D S Y G Y Q Q Q P P R P P C D P C P N A T G G G Y G V N E Y G G P A A Y N K Y N A G G G C M M S G Q Q I I P Q G Q I V R V C N K A N P D Y A L A I R D G R A V M A F Y N P N D P T M Q W V K D E S W S N Q V K D Q V G H P A F A L V N K A T G Q A L R H A I A E C Q E V L V T Q Y E G P S T Y D E N I L W S E S E D M G Y G Y R T I R M A N N I G L N L D A F Q G D K R S G G I R D G T R A V L W K W N K Q D N Q L W K L S P C Y

>PintaEULS3A

M E F P S G H H S H H H H K D H E E E R R E R E H H R H G F Q P S P E F T P P P R A G E Y Y P P P G T R F P E P E Y Y A P P P P P Q P Y S Q V E H H A H Q S P S Q G E D Y R A H Q G F G H V V H H E S N R P F Q R P E Y G T P S D S F P R P S C E T S E G P I A Q P G Y G P S R P G R K P A L P R G Q V V R V F N K A N P D F W L A I R D G S T V L V H A N P N D E S Q Q W V M D E T Y S T K V K D E A S S P S F S L V N K A T G Q A L K H G L G E T Q P V L L T Q Y D P N N F D E S I L W T T S G D M G Q G F R T I R L V N N I H L N L D A F H G D K K S G G I K D G N P V V L W S W K K G D N Q L W K I V P F

>PintaEULS3B

M E F P Y G H H S H H H K D H E E E R R E R E H H H H G F Q P S P E F T P P P R A G E Y Y P P P G T R F P E P E Y Y A P P P P P Q P Y S Q V E H H A H Q P P S Q G E D Y R A H Q G F G H V V H H E S N R P F Q R P E Y G T P S D S F P R P S C E T S E G P I A Q P G Y G P S R P G R K P A L P R G Q V V R V F T K A N P D F W L A I R D G S T V L V H A N P N D E S Q Q W V M D E T Y S T K V K D E A S S P S F S L V N K A T G Q P L K H G L G E T Q P V L L T Q Y D P N N F D E S I L W T T S G D M G Q G F R T I R L V N N I H L N L D A F H G D K K S G G I K D G N P V V L W S W K K G D N Q L W K I V P F

***Populus trichocarpa***

>PoptrEULS3A

M E F P P G H H S N T H H H R R N D D E E E R R E N Y P P P D T T P P P P S F H Q P P P P S S H Y Y Q E P P Q P P S F H Q P P P P S S H Y Y Q E S P Q P P S F H Q P P P P S S H Y Y Q E P P Q P P R P Y F Q E A T Y A P S P P P P F Q E T Q V I R T S H H Y P P P P T Q V N H V S H E K T E T H Q S F K P H M P S S I H Q Q T H Q S G S A S G L D L Y N K P S F K V Y S K A E P D F H L T I R D G R V I L S R S N P S D E F Q N W F K D E K Y S T R V K D S E G C P A F A L V N K A T G Q A I K H S I G E A N P V Q L I P Y N P D V L D Q S I L W T Q S K D L G D G F R A V R M V N N T H L N V D A F H G D K K S G G V H D G T T I V L W K W N K G D N Q R W K I I P A Q Y

>PoptrEULS3B

M E F P H G Y Y P Q T H H H R R N D E E E E R R E H Y P P P S F D Q T P P P L F Y R E N E F A P A P R P Y S H Y Y Q E S P Q P P R P Y F N E T N Y S P P P P P T S I Q E T Q V F H T S S F D Q T P P P L F Y G E N E F A P P P R P Y S H Y Y Q E S P Q P P R P Y F N E T N Y S P P P P S T S I P E T Q V F H T S H H Q G V D P S L D Y P P A P T Q V T H V S H E Q T E A R H S F R P H M P S F N H Q H T H Q P G A A S G L D L Y N K P S F K V Y S K A Q P E F H L T I R G G K V I L A P S N P S D E F Q N W Y K D E K Y S T R V K D S E G C P A F A L V N K A T G Q A M K H S I G E A H P V Q L I P Y N P D V L D E S I L W T E S K D L G D G F R A V R M V N N T H L N V D A F H G D K K S G G V H D G T S I V L W K W N K G D N Q R W K I I P T R Y

***Ricinus communis***

>RiccoEULS3

M E F P F G H Q S H T H H H H Q R N N A D D D D E S R Q F Y P P P A S A P P P P Y T R D N E F A P P P P Y F Q E P D F P P S P T R P Y Y R E N E Y A P P P P P P P V Q Q A H V Y H S S H N Q E L D S D Y Y P P R P P T Q V T H V S H E R I G T E T Q T H H S F Q P H F P S F L Q H H T H Q S G S A S G L D L S N K P T Y K V Y S K A E P N F H L T I R D G K V V L A P S D P S D E F Q N W Y K D E R Y S T R V K D E E G F P C F A L V N K A S G Q A M K H S I G G T H P V Q L I P Y N P N V L D E S I L W T E S K D L G D G Y R A V R M V N N I H L N V D A F H G D K K S G G V H N G T T I V L W K W N K G D N Q R W R I T P H

***Selaginella moellendorffii***

>SelmoEULS0

M F F F L A L V G L V S S F R S Y Q L R T K A S S A Y C L T A L N E T V R M N L C D S T C R N Q I W F L N S T Y N F V D A A G S K A F V L T N A G S G R V L R H R A N D D E Q V V L I V Y C E L S S S I L W T L S P E S Y D K Y F A I R P F E N T Q L N L D V D H G D R K H G G V Q P N N R L M L F S W H T G D N Q L W E F I E A

>SelmoEULS1A

M S Y Q Q G Y G S Y E Q H A H G H P P V Q G Q V V R V F C K A R P D F Y L T A T D D G V V L S Q G N V Q D P R Q Q W I K D D S W G E R L T D S S G S K A F A L I N K A T G Q A L R H G N S E N E Q V V L S A Y S K N E M D D D L L W S A S D D L G H G F H T V R T Y N N I D L N L D A D H G D K K H G G I K E G N K L I L F K W Q K T H D N Q F W K I V P V

>SelmoEULS1B

M Q P I Q D F P P A F D P P P P P Q Q D G A S T V R I F N K A N P S L S L T G L W N G K V V M T L G N G T M P Y Q Q W F V D T S W G T R F T D A V G Y K A F M L V N K A T G M I L R H G Y L E N S A V S C G I V K G D L V D S L W T R G P D L Q G F H T I R A C S K V E L I M D V E R A D L K K H I L L K E G C K V V M F K M S Q E E T Q Y W R M E A I V S S S

>SelmoEULS1C

M G R F L A I L C R I L L I A L I F G C V L G N G D D T W F S L E C V A S T T L R V T A F P N G H T V R L R P D D G S W N Q Q W N A T Y D D R F P G A Y S L I N R A T G L A L F H T D V A M D E V Y Q E K N E L A A A A L W V D Q P F Q G S Q L W R T I E P Y S Q Q G M V L D A F R P H H K D H L V D G N E V I I F P R A P E K Y M Q N Q V W K R N V V N S

>SelmoEULS3

M G G S E E H G G Y G Y G G Q Y Y P P P D P S I Y P P P Q G A Y G S Y P P I P S Q D R A F D A P P Y P P P S N P P N L Y P A P S Y E H D H H H H H H H D H H N H H H D H H R H E E H Q G R P D R E L G Q L V K L C C Q A N M D F S L A V R D D G V V L V P S N H H D D S Q Q W Y K D M S W S T R V R D E K G F P A F A L I N K A T R K A L K H A T E E L Q Q V L L V D Y N P R V V D E S V L W T V S E D M G S G F R T I R M A S N I K L N L D A F R G D K R S G G V K D G T P A V L Y R W K K Q E N Q L W K I I P L

>SelmoEULS4

M Y S G G G G Y Y G G E Q E E R R G G G G G Y D E G G Y G G G G Y G G G Y G G G R G E S G G G Y G G G G E Y G G G G R G E S G Y G R P E R G G Y G G G R E E E E E A Q F H R R R P P P Q S S E K D Q L V K I F C A A N P D Y V L T V N G D E L V L A P G N G S N E S Q Q F V K D C R W G S K V Q D E A G S P A F S I V N K A S G L A L Q H G S D S F E K V M L S P Y D P E R L D E S V L W T Q S D D V G G G Y Q C L R P V G N V H L N L D V K G G E G V R S G A E L I L F K W N K Q D N Q K W K I I P I E N S G G S Y G H G Y D E Q G A H G Y G E E G R H G Y G G R P A Y G D Y

>SelmoEULSv1

M K A A A V A I L L L Q L T L A P S R A E P S S S L S E A S C A E I V P R S N L T M C L T A Y A S Q G T T C A I M S P C M H K D P S Q L W I R R S N A G Y V D S H G L D A F E L I N T G S Q L A F Q H Y V D A F I T L G T V G E Q P G E Q P D L N S S L W T R Q A V S H N Y Y T L R S Y D Q P N L V V T W V S S E L P C M G S Y S H G E I N Q E W A F I N S V C

>SelmoEULSv2

M N A A A A A A A I I L V L Q F A A P A M E T E A S E L P S Y C A A Q L V P R A N L T M C V T G L S Q G V V V M S R C I R D D P Q Q L W S R S S R A G Y R D P D G R D A F V I V N L W S R L A L Q H G H E S G S E I S C G S V Q R V N S S L W T M A S A S R S Y V T L R S Y D N P E L V L D I A H G D W T H G G V R E N S F L I A H P Y N H Q E N E Q W A F I N S V C

***Sorghum bicolor***

>SorbiEULS1

M A Y N G R D Q R Y G G G I A T P G Y A P P V P Y G M S Q V N I E G N G C G R P L P P Q P T V K V Y C R A N P N Y A M T I R D G R V V L A P A N P K D E Y Q H W I K D M R W S T S I K D E E G Y P A F A L V N K A T G E A I K H S L G Q S H P V R L V P Y N P D F L D E S V L W T E S R D V G N G F R C I R M V N N I Y L N F D A L H G D K W H G G V R D G T D I V L W K W C E G D N Q R W K I Q P Y Y

>SorbiEULS3

M E F P H H H H H H G H R R D D D E D D R R G P P P P A Y G G Y G Q P P P P D P Y G R A P V D P D P Y G R P P P Q S A Y G G G G G Y G N V V H V G H E P G D G R P H Y G G G G L G G A Y G G G G P E Y G H E G R P H H H G G S E Y G H E T R P H H G A G G G G A A P V R Q Q T Y R I Y C K A G D D Q Y S L A A R D G K V C L V R T E R N D D T Q H W I K D M K Y S T R V K D E E G Y P A I V L V N K A T G E A L K H S L G Q S H P V P L T R Y D P D K L D E S V L W T E S R D V G D G F R C I R M V N N I Y L N F D A L H G D K D H G G V H D G T T L V L W E W C E G D N Q R W K I V P W

>SorbiEULSv1

M A R L T A C F A L S A V L V L A T G I S S G N G Q L L D L I P T D A T T G C L Q F P S G Q L F P S P D L L P L A Y K F L G S L P P M R I I S R G N T S L S V A A D D N G N V V L A K T N C W D A R Q L W V Q H Y P I L F D K T R F S L V N L G N K G P L L A M P I P E D S S L Y P V K L A P Y S Y S P W L F F M P V P T S M L W T Q E T P L A D G F Y K I R S Y K V P R L L L D G L Y G N V H E G T V V G A Y P A G H D D D N I L W K I E G F L S N P

>SorbiEULSv2

M A G L T A C F V L S G I L L L A T G I C S G N G Q I V E E P F P T N P V T G C L Q F R P D G H L P L T W P V P W V F E F L A K L P P L R I I S K G D P S L S I A A D D N G D V F L A K T N C S D L R Q L W V Q H F P Y P G E N R F A L V N L A K E Y E M R A P M I L E D G P Y P M K L G K Y A P L G V P A Y A L W T Q D T P L L C A G F Y K I R S C Y E D E R L V L D G L H G N V H E G T V V G A Y P A D H S A D N V L W K M E G F L S N H P

>SorbiEULSv3

M A R S T S T A A A A S G L L A A C A V L L L F A G Q S A A T T F D F P F I A I F P S V F P T G S Q S P P T V R I Y S K Q N T G L N M A V L D G K V V L V T A T D G D A K Q L W W K I L A P T W V G Y G Y W L V N V A T R Q A M A P P A Y G M Q V Q L V D F N P L I N G K Q W L W V P T A A L D G G V F Y Q I K A Y T Q Y S K A L N G L G G Y A Y D G T V I G I Y P D T P A S P N T L W I T S T D F P F R F Y

***Triticum aestivum***

>TriaeEULS1A

M D Y Y R E T Y G G Y G M A T P G Y A A P V P Y G M S Q V N I D G N C G G R P M P P Q P T V K I Y C R A N P N Y A M S V R N G K V V L A P A N P K D D Y Q H W I K D M R W S T S I K D E E G Y P A F A M V N K A T G Q A I K H S L G Q S H P V R L V P Y N P D Y L D E S V L W T E S R D V G N G F R C V R M V N N I Y L N F D A L N G D K Y H G G V R D G T E V V L W K W C E G D N Q R W K L Q P Y Y

>TriaeEULS3A

M E F P H R H G H G H G R R D D D D E D R R A P A P Y G R H E P D A Y G A P P P S Y G R R P D D D A G D A Y G R H P P A A Y G A P P P S Y G A P P P A Y G G G R E D D Y G G R A P A Y G A P A P A Y G G G R E D D Y G R H A P A P A G Y G G G D Y G R H A P A P T Y G G G R D E G Y G A P A Y G N V V H V A H E S G D E R P Q Y G G G G S G G Y G H E T R P H H G G G A A P A T R Q P T Y R I L C K A G E D S F S L A A R D G K V C L V R T D R D D D T Q H W I K D M K Y S T R V K D E E G Y P A M A L V N K A S G E A L K H S L G Q S H P V R L T R Y N P D T L D E S V L W T E S R D V G A G Y R C I R M V N N I Y L N F D A L H G D K D H G G V R D G T T L I L W E W T E G D N Q R W K I V A W

>TriaeEULSv1

M A A A A A G L V W A T L L M C A A L G A G V E N P F F P P G S E P E P V R I Y S Q E N A A L N V A E R D G N V V L A Y G D C S D L R Q K W F I V T S P H P S A P F E L P P P F V L Q N A Q T F Q V I I I P S G S G Q K V R L S S E P P I N P L D W L S W S R A A L E Q R W T P E K S T R A D H F Y Q L S V T K S D P A L T L N G L L G N V H D G T E V G I Y H A S P D S G N A I W Q M T S Y P L C A R

***Vitis vinifera***

>VitviEULS3

M E F P F G H H H H T H H H R P E Y G A G E E E E Y L Q P P P P P P L I H P P P P P P A Y F H G E D E P P P P A Y F Y G E D E P P P P R A Q V H H I S H A D P P R P P P S S H V H H I S H V E D Q G F G R E N Y T H E G P P Y A R P P P V S Y S A S A P V R H V S H H L N P Q P P Q G T E Q V E T H H L H H L P G F L H H H N E A H G V G S N L S N K P T V R V F C K A K P N H S L T I L D G K V Y L A P S D K T D M L Q H W I K D E K Y S T S V K D E E G F P S F A L V N K A T G Q A M K H S I G A S H P V Q L I P Y N P D V L D E S V L W T E S K D L G D N F R S I R M I N N I H L N V D A L H G D R S H G S V H D C T T I V L N K W K K G D N Q L W K I S L Y

***Zea mays***

>ZeamaEULS0

M A Q S V K I L C K D G F N L Y V T I R G N K A V L A P E D P D D E M Q E W F K D Y S R V S S V T D D N G Q R V F A L V N K E T Q Q A M V N R R D E Q E V Q L V P Y Y K D D D R V D I S M L W T E L P E D H G D G F H Q I A A L K N T Y E A L D G L G G N C K E G T I V G I D P T H Y S Y L G T V N K N Q L W K I I L S S A T

>ZeamaEULS2

M D G G Y Y G G R D Q R Y S G G Y Y G G G G I A T P G Y A P A V P Y G M S Q V N I E G N G C G R A L P P Q P T V K V Y C R A N P N Y A M S V R D G K V V L A P A N P K D E Y Q H W I K D M R W S T S I K D E E G Y P A F A L V N K A T G E A I K H S L G Q S H P V R L V P Y N P D F L D E S V L W T E S R D V G N G F R C V R M V N N I Y L N F D A L H G D K W H G G V R D G T D V V L W K W C E G D N Q R W K I Q P Y Y

>ZeamaEULS3A

M E F P H H H H H H G H R R D D D E D D R R G P P P P V Y G G Y G Q P P P P D P Y G R A P V D P D P Y G R P P P Q S A Y G G A G D A Y G H S G Q G Y A P P P A Y G A G G G Y G N V V H V A H E V G D E R P Y Y G G G G G G L G G A Y G G G G S E Y G H E G R P H H G G G G S Q Y G H E T R P H H G G G G G A P P V R Q Q T Y R I Y C K A G E D Q Y S L A S R D G K V C L V R T D R D D D A Q H W I K D M K Y S T R V K D E E G Y P A I V L V N K A T G E A L K H S L G Q S H P V L L T R H N P D S L D E S V L W T E S R D V G D G F R C I R M V N N I Y L N F D A L H G D K D H G G V R D G T A L V L W E W C E G D N Q R W K I V P W

>ZeamaEULSv1

M A R P S A A A A G G F L L A C A A L L L F A G Q S S A Q T T F I F P F A S L F P V G Y V P P I V R I Y S K Q N T G L N M A V R Y G K V V L V T A N S W D Q T Q L W W K I P P L T S L F D G E G A K Y W L V N V A T K Q A M T C P N G P M Q V Q L A E F N P Y N G A Q M W V P S T P R L Q D G V F Y Q I K P Y T Q D S K A L N G L G G Y V H D G T E I G I Y S D T P V S S N T L W F T S N V F P F P F Y

**Two-domain lectins**

***Hordeum vulgare***

>HorvuEULD1A

M F G F G H H H K D Q A P A A S G P N Q I F K I Y C R A S E D Y C L A V R D G E V V L A P V N P K D E T Q H W L K D M R F S T T V K D E E G M P A F A L V N K A T G L A V K H S I G Q S H P V K L V P F N P A Y E D A S V L W T E S K D V G K G F R C I R M V N N T R L G F D A L N G D K D H G G V H D G T T V V L W E W C K G D N Q C W K V W P W A E A H A A V E S G A T M G N N P H A M G G G P P V H A V R V F C K A G E D Y S L T A R N G T V C L A P T N P R D D Y Q H W I K D M R H S N K I R D E E G Y P A F A I V N K V T G E C I K H S T G Q G H P V K L V P Y N P A Y Q D E S V L W T E S R D V G K G F R C V R M V N N I Y L N F D A F H G D K A H G G V H D G T E V V L W K W C E G D N Q R W K I L P W

>HorvuEULD1B

M F G F G H H G H H G Q N P P A H A P P A A G G N Q P T F K I F C K A D E G Y C L S V R D G N V V L A P S N P R D E H Q H W F K D M R F S T Q I K D E E G N P A F A I V N K A T G L A V K H S L G Q S H P V K L V P F N P E F L D E S V M W T E S G D V G K G F R C I R M V N N I R L N F D A L N G D K D H G G V H D G T T V V L W E W A K G D N Q S W K I L P W G E E A Y A G G S A N A P R G G S S E P T V R I F C K A D E G F S A T V R N G T V V L A P G N P R D E Y Q H W F K D M R H S N R I K D E E G Y P A F A L I N K V T G E A L K H S Q G E G H P V K L V P Y N P N Y Q D E S V L W T E S R D V G A G F R C I R M V N N I Y L N F D A L H G D K E H G G V R D G T S L V L W K W C E G D N Q R W K I L P W

***Oryza sativa***

>OrysaEULD1B

M F G F G H H G H H G Q D Q P P Q H H G G G G G G A H Q P T F K I F C R A D E G Y C V A V R E G N V V L A P T N P R D E H Q H W Y K D M R F S A K I K D E E G N P A F A L V N K A T G L A I K H S L G Q G H P V K L A P F N P E Y P D E S V L W T E S G D V G K S F R C I R M L N N I R L N F D A F H G D K D H G G V H D G T T I V L W E W A K G D N Q C W K I L P W G D E A Y A G G S A N A P R G G N E P T V R I F C K A D E G F S V T V R G G S V C L A P T N P R D E Y Q H W I K D M R H S N S I K D E E G Y P A F A L V N R V T G E A I K H S Q G E G H P V K L V P Y N P G Y Q D E S V L W T E S R D V G H G F R C I R M V N N I Y L N F D A L H G D K D H G G V R D G T T V A L W K W C E G D N Q R W K I V P W

>OrysaEULD1A

M F G F G H H H N Q A P A A P S D P N Q I F K I F C R A N E N Y C L T V R D S A V V L A P V N P K D E H Q H W F K D M R F S T K V K D G E G M P A F A L V N K A T G L A V K H S L G Q S H P V K L V P F N P E Y E D A S V L W T E S K D V G K G F R C I R M V N N T R L N L D A F H G D K D H G G V R D G T T V V L W E W C K G D N Q S W K I L P W G P E A H S S S P G A A T A C T I G G V P V H T V R V F S A A G E D Y C L T V R N G T A C L A P K N P R D D Y Q H W I K D M R H S N K I R D E E G Y P A F A L V N K V T G E A I K H S T G Q G H P V K L V P Y N P E Y Q D E S V L W T E S K D V G K G F R C I R M V N N I Y L N F D A F H G D K D H G G I H D G T E I V L W K W C E G D N Q R W K I L P W

>OrysaEULD2

M F S H H G H G H G Q Y Q P P A T G P Q H E P T F K I F C R A D E G Y C L T V R H D A V V L A P T N P R D D C Q H W Y K D M R H S T R V K D E E G H P A F A L V N R A T G L A V K H S L G Q S H P V K L V P Y N P E Y Q D E S V L W T E S K D V G H G F R C I R M V N N I Y L N L D A F H G D K S H G G V H D G T T V V L W E W C K G D N Q C W K I L P W G P E A Y A P P P P P A Y G H Q A Y P P P P P N R E P G H G Y H P A P A F Y P P Q P P P S H D E P G Y G Y R P P P V G P P G A G Y G N R L P R A L A S E P T V R I L C R A D E A Y S L T V R N G A V C L A P T N P R D D F Q H W V K D M R H S T S I K D E E G Y P A F A L V N K A T G E A I K H S L G Q S H P V R L V P Y N P E Y L D E S V L W T E S K D V G H G F R C V R M V N N I Y L N F D A F H G D K D H G G V H D G T T V V L W E W C K G D N Q R W K I L P W

***Picea sitchensis***

>PicsiEULD1

M H N P F G H H Q P E P Q Q P Y Q A P H G G Q Y P P P I Q G E T V K I Y C E A N P D F L L A S R N E S V V M V P A N E S D P S Q Q W I M D T S W S V K A K D D A G F P A F A L V N K A T G Q A L R H G R S E K D K V T L G P Y H P D D L N E A V L F T Q S A D V G K G Y Q C I R P V N N T H L N L D A S A G D D K H H V A L S E G T E I V L C K W N K K E S Q K W K I S P I L G S S H G A Y P P R G E P S S F F P Q A N V E P Q G S T V R I H C E A N P E F F L A A R G D V A V L A P E N P R D P H Q Q W I K V D S W G L R V K D E A G F P A F A L V N K A T R H A L K H G D K E W D Q I Y L A D Y N H D K A E Q S V L W T L S A D V G N G Y Q C L R P V N N I N L N M D A K A A D G H V G G I R D G N E L I L F E W K K Q S N Q K W K L Q P V H

>PicsiEULD2

M F H H Q K E V E A P G E I V K I Y C E A N P E Y Y L T A K E D S V V L A P G D E S N P Y Q Q W I I D G T W G I R V K D S A G F P A F A L I N K A T R Q A L R H G K E E K E K V H T C P Y S K D E L N E V V L W T Q S D D V G N G Y K C I R P V D N T D L N L D A N H G D P E S G G I Q D G T D L I L F K W K K Q E N Q K W K I L P I S F N P I S Q V Q K Y E A E D T P Y G G V P K Y G G V S S Y E G A Q Y G E D V Q S Y G G A S H G K D V P S Y G S A P Y G E G V P N Y E G A P Y A E G V P N Y E G A P Y G E R T D K V T N Y E G P A Y A V H T H H H H H D F P Q H I P E G Q S V R I Y C E Q S P E Y F L T V R D G E V V L T P G D A D D V S Q H W I K V D E W G N K I R D E V G F P A F A L V S K A T L K A L K H G S Q E W D R V E L S Y Y N E N D L D E S V L W T Q S A D V G R G Y Q C I R P V G N I H L N I D A K L A D G K H G N V E D G N E L I L F S W K K Q K N Q K W K M L P V D

***Pinus taeda***

>PintaEULD1

M H N P F G H H H P E P Q Q P Y Q A P H G S Q Y P Q P I Q G E I V K I Y C E A N P D F F L A G R N G S V V M A P A N E S D P S Q Q W I M D T S W S V K A K D D E G F P A F A L V N K A T G Q A L R H G R A E K D K V T L G P Y H P D D L N E A V L F T Q S A D V G K G Y Q C I R P V H N T H L N L D A S A G D D K H H V A L R E G A E I V L C K W N K K E S Q K W K I S P I L G A S Q G V H P P R G E Q S S F Y P P Q A N V E P Q G H T V R I H C E A N P E F F L A A R G D V A V L A L E N P H D P H Q Q W I K V D S W G L R V K D E A G F P A F A L V N K A T R H A L K H G D K E W D Q I F L A D Y N H D K A E Q S V L W T L S A D V G N G Y Q C L R P V N N I N L N M D A K A A D G H V G G I R D G N E L I L F E W K K Q S N Q K W K L Q P I H

>PintaEULD2A

M F H H H N E D E T P G E I V K I F C E A N P E Y Y L T A K E D D V V L A P G D E S N P Y Q Q W I I D A T W G L R V K D E A G F P A F A L I N K A T R Q A L N H G K E E K D K V Y T C P Y S K D E L N E V V L W T Q S N D V G N G Y K C I R P V D N T D L N L D A N H G D P E S G G I Q D G T D L I L F K W K K Q E N Q K W K I S P I S F N P I S Q V Q K H E A E D A P Y G D V P K Y G G A Q Y G D V P S Y G S A S Y G E G V P S Y E G V P N Y E G A P Y G E R T D T V T N Y E G P A Y A V Q V H H H H D D F P Q R I P E C Q S I R I Y C E Q S P E Y F L T V R D G A V V L A S G D A D D V S Q H W I K V D E W G N K I R D E A G F P A F A L V N K A T L K A L K H G S Q E W D R V D L T Y Y N E N D L D E S I L W T Q S A D V G H G Y Q C I R P V S N I N L N L D A K L A D G K Y G N V E D G N E L I L F S W K K Q K N Q K W K M L P V D

>PintaEULD2B

M F H H H N E D E T P G E I V K I F C E A N P E Y Y L T A K E D D V V L A P G D E S N P Y Q Q W I I D A T W G L R V K D E A G F P A F A L I N K A T R Q A L N H G K E E K D K V Y T C P Y S K D E L N E V V L W T Q S N D V G N G Y K C I R P V D N T D L N L D A N H G D P E S G G I Q D G T D L I L F K W K K Q E N Q K W K I S P I S F N P I S Q V Q K H E A E D A P Y G D V P K Y G G A Q Y G D V P S Y G S A S S G E G V P N Y E G A P Y G E R T D T V T N Y E G P A Y A V Q A H H H H D D F P Q R I P E G Q S V R I Y C E Q S P E Y F L T V R D G T V V L A S G D A D D V S Q H W I K V D E W G N K I R D E A G F P A F A L V N K A T L K A L K H G S Q E W D R V D L T S Y N E N D L D E S I L W T Q S A D V G H G Y Q C I R P V S N I N L N L D A K L A D G K Y G N V E D G I E L I L F S W K K Q K N Q K W K M L P V D

***Physcomitrella patens***

>PhypaEULD1

M Y H G E D A R S N N P Y S D Q G F G H G G G Y E P S R R R Q L P G E A V R L H C R A D P N F C L A A I P G Q G P V M V P N N E S D A Y Q V W Y K D E S M S N R V K D E S G A H A F S L I N K A T G E C L R H P P E D L Q Q C L L V V Y E P N A Q D E S V L W T M S E D M G Q G Y R C I R V V T S I T R N L D V L R G D K K S G G M K N G S S V I T F A W K N Q D N Q V W K M T P V G P G I G G S A A Q P S I Y P S A P G T G V G V S A D Y E P P R R R Q L P G H A V R L H C R A D P N F S I A V I P G Q G T I M V P T N A S D A H Q I W Y R D E S M S N R V T D E S G A H A F A L I N K V T G E C L R H P P E D L K Q C L L A D Y E P N G L D E S V L W T M S E D M G Q G Y R C I R V V T S I T R N L D V L R G D K K S G G V K T G S P L I T F A W K N Q D N Q V W K M T P A

***Selaginella moellendorffii***

>SelmoEULD0

M P Q G Q F V K I Q C K A D S A Y N L A A R T D G V V M A P A D E S D P R Q Q W Y K D E S W E H V K D A K G S A S F A L I N R A T G Q A L T H P G G L D T Q V T M S D Y F G D S E D K S T L W T L S Q D V C G G W N A L R P Y Y D T S L N L S T N H W D K K H G G L T D G T D V L I T K W K E Y D N Q C W K L V P T R D S T G G A D H F H R V L R T E S R V V D R S L H H L P K Y S L V T K A G E G Y S V S A R G D G V V L A P S D P S D G R Q Q W I K D D W W G S K L L D S L G Q P A F V M L N V A T Q H A L S H A P G E G Q Q V S L A K F V P N L L N Q D I L W T K Q E V G H G W F A V R P A N N V K L T M D A F R A D K K H G G P S D G T K V V V F K W S D Q E N Q H W K F T E

>SelmoEULD3

M A E Y G G Y G G A P D T T A A P Y G S G P E Y S S Y G G G R M D E S E P R G F F H H D K K P G H E H H H E Q P H R F D Q E N V G G G Y G E Q Q Q Q S G Y H S R P G G G G G S A H E E V F K I V C E A N P S Y Y L T D R G G E L V L A P G N E S N P H Q Q W Y K D T R H S S K K D K E G F P G F S V I N K A S G L A L Y H D D K K D K V Y L K P Y D A N D T S E S F L W S Q S A D V G H G Y Q C I R P F T D V H L N L D A Y R G D K E H G G V K D G T T V I L H K W N K Q E N Q K W K I L P I T S T G A Y G A E A Q S Y G T S A A Y G G G Q G Q S Y G Q Q T V K L S C E A R P D Y Y V T V R G D S V V L A A S E E S D P R Q E W F Q D N K L A A N M K D E S G S P A F V L I N K A T Q K A L Q H G E K E F D K V R V V S L H G D R P D E S V L W T L S A D V G K G Y R C L R P V G N I H L N L D A S H G D E K H G G V H D G T E L I L F K W L K R E N Q K W R L T Y L

>SelmoEULD4A

M A L T A A P K F F T I E S A A Y P G Y C I T Q Q G N Q V V L K S K D A L N P F Q S W N L L N Q E L S G R D E F L D P D I S H L S A F V F V N H G S K K A L R S Q A Y S T S Q S K A T I G D Y D P V G Q D N Y L L W R I S G S H E N Q V L I T M T V N K S S F L S H T Y S S A S F K D G V P V V T S N S N E N S H W K L V T Y E P S Q E Y R I L C H W F G Q Y L T W K Q Q N V D V T F K N A V K H N D E T N V V A I S P N N V P L D G M T W Y K V P A G G R D D T T Y F T S F V L I N K V T G L A L K G S A K P G D K V L L T Q Y D P L N Y S F V W S E N R F Q A S N T D Y L A I N L D G T N L Q W A L Q N D D Q Y Q N S A W Y H P L V L R S A S P N Q G Y L E Q Q A W K F N P V V A G N

>SelmoEULD4B

M A L T A A P K F Y T I E S A A Y P G Y C I T Q Q G T Q I V L R S K D A L N P F Q L W Y R D E S W N L L N Q D S A F V F V N H G S K K A L R S Q A H S T S R S K A T I G D Y D P A V Q D D Y L L W R I S G S H E N Q V L I T M T G N K S F F L S H T S S S A S F K D G V P V V T S N S D G N S H W K L V T Y E P S Q E Y R I L C H W V G Q Y L T W K Q Q N V D V T F K N A I K H N D E T N V V A I H P S N V P L D G M T W Y K V P A G G R D D T T Y F T S F V L I N K V T G L A L K G S T K P G D K V L L T Q Y D P L D Y G F V W S E N R F Q A S L T D H L A I N L D G T N L Q W A L Q N D D Q Y Q N S A W Y H P L V L R P A S A N Q V F N T Q Q A W K F N P V V A G N

>SelmoEULD4C

M E P L T A V P K L Y A I E S T A Y P G Y C I T Q Q G T Q I V L K F K D A L N P F Q L W Y R D E S W Q F L S E D N S D L S A L V F I N H G S K K A L R S Q S S S T S R S I A K L T V G D Y D P A V E N N S L L W S I S G S S D D Q V F V T E T G N S S V F L S H T S S S R S S F K D G V P V V T S A Q G E N S R W K I V A Y V P S Q V Y R I L C H W F G N Y L T W K Q Q N V D V T F K N A I K H N D E T N V V A V S P N N I S L D G M A W F K V P A G G R D N T T Y F T S F I L I N K T G L A L K G S T K P G D K V L L T Q Y D P L D Y G F V W S E N R F Q A S N T D Y V A I N L D G T N L Q W S L Q N D D Q Y Q N S A W Y H P L V L R P A S A N Q G Y V E Q Q T W K F N P V V N N V

>SelmoEULD4D

M A L T A A P K F Y T I E S A A Y P G Y C I T Q Q G T Q I V L R S K D A L N P F Q L W Y R D E S W N L L N Q E F R D S S A F V F V N H G S K K A L R S Q A H S T S R S K A T I G D Y D P A V Q D D H L L W R I S G S H E N Q V L I T M T G N K S S Y L S H T S S S A S F K D G V P V V T S N S D G N S H W K L V A Y E P S Q E Y R I I C D W Y E Q Y L T W K Q Q N V D V T F K N A I K H N D E T N V V A I S P N N V P L D G M T W Y K V P A G G R D D T T Y F T S F V L I N K V T G L A L K G S T K P G D K V L L T Q Y D P L D Y G F V W S E N R F Q A S K T D Y L A I N L D G T N L Q W S L Q D N N Q Y Q N S A W Y H P L V L R P A S A N Q G F V Q Q Q A W K F N P V V A G N

>SelmoEULD4E

M A L T A A P K F Y S I E S V A Y P G Y Y V T Q Q G T Q I V L K S K D A L N P F Q L W Y R D E S W Q F L T Q E I S D L S A F V F I N H G S K K A L R S Q E I A T S R H F A K A T V R D Y D P A V E D D S L L W S V S R S S D D Q V S I T M T G N S S V F L S H T S S S S N F K D G V P V V T S T S G G S S R W K L V T Y E P S Q V Y R I L C H W F G Q Y L T W K Q Q N V D V T F K D A I K H N D E T N V V A V S P N N I S L D G M I W Y K V P A G G R D D T T Y F T S F I L I N K V T G L A L K G S T K P G D K V L L T Q Y D P L D Y G F V W S E N R F Q A S N T D Y L A I N L D G T N L Q W A L Q N D D Q Y Q N S A W Y H P L T L R P A S A N Q G Y L E Q Q A W K F N P V V A G S

>SelmoEULD4F

M A L T A A P K F Y T I E S A A Y P G Y C I T Q Q G T Q I V L R S K D A L N P F Q L W Y R D E S W N L L N Q D S A F V F V N H G S K K A L R S Q A H S T S R S K A T I G D Y D P A V Q D D Y L L W R I S G S H E N Q V L I T M T G N K S F F L S H T S S S A S F K D G V P V V T S N S D G N S H W K L V T Y E P S Q E Y R I I C D W Y E Q Y L T W K Q Q N V D V T F K N A V K H N D E T N V V A I H P S N V P L D G M T W Y K V P A G G R D D T T Y F T S F V L I N K V T G L A L K G S T K P G D K V L L T Q Y D P L D Y G F V W S E N R F Q A S K T D Y L A I N L D G T N L Q W A L Q N D N Q Y Q N S A W Y H P L V L R P A S A N Q A F G T Q Q A W K F N P V V A G N L S A T

***Sorghum bicolor***

>SorbiEULD1A

M S W F G H H H H N Q P A P P A S G P N Q V F K I F C R A N E N Y C L A V R D G A V V L A P T N P K D E H Q H W Y K D M R F S T R V K D E E G M P A F A L V N K A T G L A I K H S L G Q S H P V K L A P F N P D Q E D A S V L W T E S K D V G K G F R C I R M V N N T R L N F D A F H G D K D H G G V H D G T G V V L W E W C K G E N Q S W K I L P W G P E A N A A A G A N G G V H T V R V F C K A G G E D Y C L T V R N G T A C L A P T N P R D E Y Q H W I K D M R H S T R V R D E E G Y P A F A L V N K V T G E A L K H S T G Q G H P V K L V P Y N P E Y Q D E S V L W T E S R D V G N G F R C V R M V N N I Y L N F D A F H G D K A H G G V H D G T E I V L W K W C E G D N Q R W K I L P W

>SorbiEULD1B

M F G F G H H G H H H G E N P P A H P P A H G G V H P P T F K I F C K A D E G Y C L T V R D G N V V L A P S N P R D E H Q H W Y K D M R F S N Q I K D E E G N P A F A L V N K A T G L A I K H S L G Q S H P V K L V P F N P E Y Q D E S V L W T E S G D V G K G F R C I R M V N N I R L N F D A F H G D K D H G G V H D G T T V V L W E W A K G D N Q S W K I L P W G D E A Y A G G S G G A A A N A P Y G H G E P T V R I Y C K A D D G F S V T V R N G S V C L A P T N P R D E Y Q H W V K D M R H S N R I K D E E G Y P A F A L V N R V T G E A I K H S Q G E G H P V K L V P Y N P N Y Q D E S V L W T E S R D V G H G F R C I R M V N N I Y L N F D A F H G D K D H G G V R D G T A V V L W K W C E G D N Q R W K I V P W

>SorbiEULD1C

M Y D Y G L G H P W L N P E G T F K V F C K A D E G L C L A V R G G S L V L A A A D P A D E H Q H W F K D V R F S L R I K D E E G K P V F S L I N K A T G L A I R H S L G P Y H P V R L L E F D P E G F E E S L L W T E S G H L G R D F G C I R M M H D V D M G L D A A L P R G E D G V R D G T G I T L T E G A E A E G D T R S W K I V F W S E E A N K T C A G L Y A E P T C R I Y C K A D E G F S L T V R D G A V C L A P T D A D D D Y Q H W I Q D K R P G N R I K D L E G Y P A F V L V N R V T G D A M D A S V G R G N P L K L K A Y N P C Y L D E F I M W T T N R D M G G G F R C I H M L C N L S L N F N A L K V D G V Q D D T K L V L S Y W G V E S D N P H L Q W K I V P W

>SorbiEULD2

M F G H H H H H G H G G H G Q A P P P P P P H E Q T L Y K I F C R A D E A Y C L T V R N D T V V L A P T N P R D E S Q H W Y K D M R H S T K V K D A E G Q P A F A L V N R A T G L A I K H S L G Q S H P V K L V P Y I P D Y L D E S V L W T E S H D V G K G F R C I R M V N N I H L N F D A F H G D K D H G G V H D G T T V V L W E W A K G P N Q S W K L L P W G D E A Y N A P R A P P G G P Y P P P A A Q P E P A Y G G Y R P P P G G P A A G G Y A P P P P G P Y P P P A P Q Q E P G Y G G Y R P Q P P V H E P G Y G G Y P P A P G N P A P G Y G P P A G Y G Y G N L P R V L A S E P T V R V F C K A G D G Y S L T V R N G T V C L A P T N P R D E F Q H W V K D M R H S T R I K D E E G Y P A F A L V N K V T G E A I K H S L G Q S H P V R L V P Y N P E Y V D E S V L W T E S R D V G H G F R C V R M V N N I Y L N F D A F H G D K D H G G V H D G T T V V L W E W A K G D N Q R W K I L P W

***Triticum aestivum***

>TriaeEULD1A

M F G F G H H H K D Q A P A A S G P N Q I F K I Y C R A S E D Y C L A V R D G E V V L A P V N P K D E T Q H W L K G M R F S T T V K D E E G M P A F A L V N K A T G L A V K H S I G Q S H P V K L V P F N P A Y E D A S V L W T E S K D V G K G F R C I R M V N N T R L N F D A L N G D K D H G G V H D G T T V V L W E W C K G D N Q C W K I W P W A E A H A A V E S G A T M G N N A H A M G G G P P V H A V R I F C K A G E D Y S L T A R N G T V W L A P H N P R D D Y Q H W I K D M R H S N K I R D E E G Y P A F A I V N K V T G E C I K H S T G Q G H P V K L V P Y N P A Y Q D E S V L W T E S R D V G K G F R C V R M V N N I Y L N F D A F H G D K A H G G V H D G T E V V L W K W C E G D N Q R W K I L P W

>TriaeEULD1B

M F G F G H H G H H G Q N P P A H A P A A A G G N Q P T F K I F C K A D E G Y C L S V R D G N V V L A P S N P R D E H Q H W F K D M R F S S Q I K D E E G N P A F A I V N K A T G L A V K H S L G Q S H P V K L V P F N P E Y L D E S V M W T E S S D V G K G F R C I R M V N N I R L N F D A L N G D K D H G G V H D G T T V V L W E W A K G D N Q S W K I L P W G E E A Y A G G S A N A P R G G S S E P T V R I F C K A D D G F S A T V R N G A V V L A P T N P R D E Y Q H W F K D M R H S N R I K D E E G Y P A F A L V N K V T G E A I K H S Q G E G H P V K L V P Y N A N Y Q D E S V L W T E S R D V G V G F R C I R M V N N I Y L N F D A L H G D K E H G G V R D G T S L V L W K W C E G D N Q R W K I L P W

***Zea mays***

>ZeamaEULD1A

M S W F G H H H H N Q P A P P A S G P N Q V F K I F C R A N E N Y C L A V R D G A V V L A P V N P K D D H Q H W Y K D M R F S T R V K D E E G M P A F A L V N K A T G L A I K H S L G Q S H P V K L A P F S P D Q E D A S V L W T E S K D V G K G F R C I R M V N N T R L N F D A F H G D K D H G G V H D G T A V V L W E W C K G E N Q S W K I L P W G P E A N S S A A H A G G P H A V R V F C K A G G E D Y S L T V R N G T A C L A P T N P R D E Y Q H W V K D M R H S T R V R D E E G Y P A F A L V N K V T G E A L K H S T G Q G H P V K L V P Y N P D Y Q D E S V L W T E S R D V G N G F R C V R M V N N I Y L N F D A F H G D K A H G G V H D G T E I V L W K W C E G D N Q R W K I L P W

>ZeamaEULD1B

M F G F G H H G Q N P P A H G G V H Q P T F K I F C K S D E G Y C L T V R D G N V V L A P A N P R D E H Q H W Y K D M R F S T Q V K D E E G N P A F A L V N K A T G L A I K H S L G Q S H P V K L V P F N P E Y Q D E S V L W T E S G D V G K G F R C I R M V N N I R L N F D A F H G D K G H G G V H D G T T V V L W E W A K G D N Q S W K I L P W G D E A Y A A G G S S A A N A P R F G G G G E P T V R I Y C K A D E G F S A T V R N G A V C L A P T N P R D E Y Q H W I K D M R H S N S V K D E E G Y P A F A L V N R V T G E A I K H S Q G E G H P V K L V P Y N P G Y Q D E S V L W T E S R D V G H G F R C V R M V N N I Y L N F D A F H G D K D H G G V R D G T N I V L W K W C E G D N Q R W K I V P W

>ZeamaEULD2

M F G H H H H H G H G G H H Q A P P P H E Q T L F R I F C R A D E A Y C L T V R H D A V V L A P T N P R D E Y Q H W Y K D M R H S T K V K D A E G Q P A F A L I N R A T G L A I K H S L G Q S H P V K L V P Y N A E Y L D E S V L W T E S H D V G K G F R C I R M V N N I H L N F D A F H G D K D H G G V H D G T T V V L W E W C K G P N Q S W K V L P W G D E A Y A P R P Y A P P P P A Q V E P G Y G G Y R P P A A G G Y A P P P P P A Q V E P G Y G G Y P P P P A A G G Y A P P P P P A Q V E P G Y G G Y P P P P A A G G Y A P P P P P V P V Q E P G Y G G Y R P P S A G G Y P A G Y G Y S N L P R A L A S E Q T V R V Y C K A G E G Y S L T V R N G T V C L A P T N P R D E F Q H W V K D M R H S T S I K D E E G Y P A F A L V N K V T G E A I K H S L G Q S H P V R L V P Y N P E Y V D E S V L W T E S R D V G H G F R C V R M V N N I Y L N M D A F H G D K D H G G V H D G T T V V L W E W A K G D N Q R W K I L P W
