# Supplementary material for: Proteins with an Euonymus lectin-like domain are ubiquitous in Embryophyta
Source: BMC Plant Biol. 2009 Nov 23;9:136. doi: 10.1186/1471-2229-9-136 (PMC2788552; doi:10.1186/1471-2229-9-136)
Supplement: Additional file 2 — Additional data from transcriptome analyses. [file 1471-2229-9-136-S2.DOC]

**Additional file 2**

**Additional data from transcriptome analyses**

To further corroborate the presence and composition of the EUL gene complement in other plant species, a thorough analysis was performed of available transcriptome data, taking advantage of information obtained from completed genomes. This section provides an overview of the most important findings from transcriptome analyses. For practical reasons the results are discussed per major taxonomic group (following the NCBI taxonomy browser) starting with the most primitive one.

***Marchantiophyta* (liverworts)**

At present, data are only available for the liverwort *Marchantia polymorpha*. It expresses two single-domain EULs that have not been identified in any other plant species. Both proteins comprise a short N-terminal domain followed by an EUL domain and a short (MarpoEULS5a) or medium long (MarpoEULS5b) C-terminal domain (Figure 3).

***Bryophyta* (mosses) and *Lycopodiophyta* (clubmosses)**

Apart from *Physcomitrella patens* no EST sequences encoding EUL proteins were deposited for any other moss. A few EST sequences encoding EUL proteins were identified in *Selaginella lepidophylla*, and confirmed what had already been observed in *S. moellendorffii*.

***Filicophyta* (ferns)**

EST encoding proteins with an EUL domain were deposited for *Ceratopteris richardii* and *Adiantum capillus-veneris*. In the former, one S1-type and two S3-type EUL proteins are expressed, but there are no indications for two-domain proteins. *Adiantum capillus-veneris* expresses an S1, an S2, and most probably also an S3 type protein.

***Cycadophyta* (cycads)**

Within the cycads, expressed proteins with EUL domains were identified for *Cycas rumphii* and 3 *Zamia* species (*Zamia fischeri, Zamia vazquezii*, and *Zamia furfuracea*). *C. rumphii* expresses at least 4 different EUL proteins. Complete contigs could be assembled for an S3 and a D1-type protein. The other two sequences are too fragmentary for unambiguous identification. All three *Zamia* species express a D1-type EUL protein. Furthermore there is some evidence that *Z. vazquezii* expresses an S3-type protein and a second type of two-domain protein.

***Gnetophyta***

Analysis of the *Welwitschia mirabilis* transcriptome allowed to identify two different forms of a typical S3 EUL protein and one D1-type protein. In addition, another unidentified form is expressed. For *Gnetum gnemon* complete sequences could be assembled for only two different S2-type EUL proteins. However, closer investigation suggested the additional expression of an S3-type protein and at least one two-domain protein.

***Ginkgophyta***

Screening of the *Ginkgo biloba* EST database returned only 5 different incomplete sequences. Aligning these with orthologs from *Pinus* sp. indicated that *Ginkgo biloba* tissues express two S2, one S3, one D1 and one D2-type EUL.

***Coniferophyta***

*Pinus taeda* (loblolly pine) expresses a complex set of EUL proteins comprising S2, S3, D1 and D2-type proteins. Five S2-type EULs (S2A-E) are expressed. These five S2-types differ from each other in length. The deletions/insertions are exclusively located in the N-terminal domain, while the EUL domain is virtually identical in all 5 subtypes. Four subtypes occur in multiple forms, varying at one or a few positions (e.g. PintaS2Ba-c). Besides S2-type proteins *P. taeda* expresses a whole set of S3-type proteins, four of which closely resemble the S3 found in other species whereas four others possess an ‘aberrant’ cysteine-rich N-terminal domain fused to a ‘normal’ EUL domain (referred to as type S3’). It should be mentioned here that orthologs of the *Pinus* S3’-type EULs were not found in species other than *Pinus*. In addition to the single-domain proteins, *P. taeda* expresses three different two-domain EULs (PintaEULD1, PintaEULD2A, and PintaEULD2B).

Even though only limited information could be obtained due to the low number of available sequences, all evidence suggests that similar to *P. taeda* other *Pinus* species also express a very similar set of EUL proteins.

Although the complement of EUL proteins expressed in *Picea sitchensis* (Sitka spruce) resembles that in *P. taeda* it is certainly not identical. Four different types of S2 proteins were identified, two of which occur in *P. taeda* as well, whereas no genuine orthologs could be found for the other two. Conversely, some of the S2-type proteins expressed in *P. taeda* were absent from *P. sitchensis*. Four S3 but no S3’ sequences were retrieved from *Picea sitchensis*. Apart from the single-domain proteins, two two-domain proteins are expressed in *P. sitchensis* (PicsiEULD1 and PisciEULD2).

***Magnoliophyta (flowering plants)***

*Basal Magnoliophyta*

From the EST databases of *Amborella* *trichopoda* and *Nuphar advena* a few EUL protein sequences were retrieved. Although incomplete, they seem to correspond to S3-type proteins.

*Magnoliids*

Transcriptome data of *Liriodendron tulipifera*, *Persea americana*, and *Saruma henryi* indicate that all 3 species express S3-type EUL protein(s). In *Liriodendron tulipifera* at least 2 distinct forms are expressed. No Magnoliid sequence is complete. Since the size of the EST databases is rather small no conclusions can be drawn with respect to the EUL protein complement of Magnoliid species.

*Eudicotyledons*

Within the stem eudicotyledons, sequences encoding EULs were identified in the EST databases of 2 Ranunculaceae (*Adonis aestivalis* and *Aquilegia formosa* x *Aquilegia pubescens*) and 2 Papaveraceae (*Papaver somniferum* and *Escholzia californica*) species. Complete sequences are available only for the *Aquilegia* hybrid which apparently expresses 5 S3-type proteins with identical EUL domains but slightly different (by short deletions/insertions) N-terminal domains. The yet incomplete sequences from the three other stem eudicotyledons almost certainly correspond to S3-type proteins.

ESTs encoding EUL proteins could be retrieved from virtually all EST databases from core eudicotyledons. Most species express a single S3-type protein but there are also several species in which two or more closely related S3-type proteins are found. In most of these cases the presence of multiple forms can be ascribed to polyploidy (e.g. potato, kiwi), indicating that dicotyledons express in general a single S3 protein per diploid genome. However, there might be some exceptions (as is illustrated by the *Populus trichocarpa* genome). Some species express a (complex) set of multiple - but all single-domain - EUL proteins. In all these cases the complement of expressed EUL proteins comprises a typical S3 protein and one or more types of paralogs with a different, shorter N-terminal domain. Despite resemblance to S1 and S2-types in grasses and Coniferophyta, the dicot proteins cannot be considered genuine orthologs of these but most probably represent S3 paralogs that evolved at different, independent occasions within the eudicotyledons.

Unlike the majority of dicotyledons, most Asteraceae species (for which transcriptome data are available) apparently express complex mixtures of EUL proteins. A fairly accurate survey of expressed EUL proteins could be assembled for *Lactuca* species. *Lactuca* species express (complex) mixtures of proteins with a single EUL domain. No two-domain sequences could be retrieved. The composition of the EUL protein complement varies with the species, yet these variations most likely do not reflect interspecific differences in the gene family but rather the fact that the transcriptomes are far from complete. Three major types of EUL proteins are expressed in *Lactuca* sp. namely S0, S2, and S3. For the S2 and S3 types at least two distinct subtypes occur. Sequence alignments indicate that the S0 and S2 types might have evolved from an S3 type by progressive shortening of the N-terminal domain. A very similar complement of EUL proteins is expressed within the genus *Helianthus* but no accurate overview could be elaborated for this genus as there were not enough complete sequences. EST data are also available for *Carthamus* sp., *Centaurea* sp., *Cichorium* sp., *Gerbera* hyb., *Guizotia abyssinica*, *Senecio* sp., *Stevia rebaudiana*, and *Taraxacum* sp. However, the data are too fragmentary for a comparison with the *Lactuca* and *Helianthus* species.

Within the Asterids EUL proteins aberrant from the classical S3 type were identified in two Plantaginaceae sp. (*Plantago major* and *Antirrhinum majus*) and in the genus *Ipomoea* of the family Convolvulaceae. *Antirrhinum majus* expresses an S3-type and at least two distinct S2-type proteins, as do both *I. nil* and *I. batatas*. In the latter two cases it also seems likely that the S2 types evolved from an S3 type by curtailing of the N-terminal domain. Two sequences identified in an EST analysis of *Plantago major* vascular tissue (annotated as lectin-like protein 1 and 2) correspond to S0-type protein. All EUL sequences found in species of the Rosids (e.g. *Prunus* sp. and *Malus* sp.) correspond to typical S3 proteins except the *Euonymus europaeus* (Celastraceae) lectin itself and an S2-type protein that co-occurs with a typical S3 in *Hevea brasiliensis*.

*Liliopsida*

A search for EUL domains in Liliopsida (monocotyledons) revealed that all EST databases from monocots contain sequences encoding EUL domains. Since apart from Poaceae species only a relatively small number of sequences have been deposited, no definitive conclusions can be drawn with respect to the EUL protein complement in other monocots.

Within the Poaceae BEP clade (Bambusoideae, Ehrhartoideae and Pooideae), transcriptome data are available for more than 10 species (*Oryza sativa*, *Agrostis stolonifera*, *Avena sativa*, *Brachypodium distachyon*, *Festuca arundinacea*, *Lolium multiflorum*, *Puccinellia tenuiflora*, *Aegilops speltoides*, *Elymus wawawaiensis*/*Elymus lanceolatus*, *Hordeum vulgare*, *Leymus cinereus* x *Leymus triticoides*, *Pseudoroegneria spicata*, *Secale cereale*, and *Triticum aestivum*). Even with limited available data for several species, all evidence suggests that they all share the same set of EUL proteins as identified for *O. sativa*. Moreover, there is also evidence for the expression of some different types of EUL proteins. First, various species express the S0-type proteins. In two species (*Leymus triticoides* and *Elymus wawawaiensis*/*Elymus lanceolatus*) a single S0 protein could be identified, whereas at least three distinct forms are expressed in *Pseudoroegneria spicata*. Second, ‘vacuolar’ forms are expressed in at least four different species (*Elymus wawawaiensis*/*Elymus lanceolatus*, *Hordeum vulgare*, *Triticum aestivum*, and *Pseudoroegneria spicata*).

In the PACCAD clade (Panicoideae, Aristidoideae, Centothecoideae, Chloridoideae, Arundinoideae, Danthonioideae) next to *Z. mays* and *S. bicolor*, transcriptomes are also available for *Aeluropus littoralis, Pennisetum sp.*, *Panicum virgatum,* and *Saccharum officinarum*. Owing to the low number of sequences no conclusions can be drawn for the former two. *Panicum virgatum* and *S. officinarum* express a set of EUL proteins similar to that of *Z. mays* (i.e. S0, S2, S3, D1, D2, and Sv). *S. officinarum* apparently expresses a very complex set of at least 21 different S3-type proteins that share the same EUL domain but differ by short insertions/deletions in their N-terminal domain. Most probably the different S3 proteins are encoded by orthologous genes of the individual genomes in the highly polyploid sugar cane.

EUL sequences were also retrieved for Liliopsida other than Poaceae species (*Acorus americanus, Zantedeschia aethiopica*, *Allium cepa*, *Asparagus officinalis*, *Phalaenopsis equestris* and *P. violacea*, *Yucca filamentosa*, *Elaeis oleifera* and *E. guineensis*, *Curcuma longa*, *Zingiber officinale*, and *Musa acuminata*). Apart from *A. cepa*, which expresses a set of EUL proteins very similar to the five proteins found in *O. sativa*, only scarce information could be obtained for the other species. Nevertheless, the whole of retrieved sequences indicates that most if not all of these monocots express a set of single-domain and two-domain proteins comparable to those found in *O. sativa*.
