# Supplementary material for: Proteins with an Euonymus lectin-like domain are ubiquitous in Embryophyta
Source: BMC Plant Biol. 2009 Nov 23;9:136. doi: 10.1186/1471-2229-9-136 (PMC2788552; doi:10.1186/1471-2229-9-136)
Supplement: Additional file 3 — Table S1 and S2. Table S1: List of metazoan EST sequences encoding proteins with an EUL domain. Table S2: Overview of sequences encoding single (S)- and double (D)-domain EULs used to construct a phylogenetic tree (Figure 6). Sequences for all EUL proteins are shown in Additional file 1: Figure S5. v: vacuolar EUL homologs. [file 1471-2229-9-136-S3.DOC]

**Additional file 3**

**Table S1**: List of metazoan EST sequences encoding proteins with an EUL domain

Description Score E value

(Bits)

[gb|DW221154.1|](http://www.ncbi.nlm.nih.gov/entrez/query.fcgi?cmd=Retrieve&db=Nucleotide&list_uids=84349728&dopt=GenBank&RID=AU1U5ZE6016&log$=nucltop&blast_rank=1) EST37412 Larval Stage 1 Aedes aegypti cDNA clo...  [228](http://blast.ncbi.nlm.nih.gov/Blast.cgi#84349728%2384349728) 3e-58

[gb|EB100634.1|](http://www.ncbi.nlm.nih.gov/entrez/query.fcgi?cmd=Retrieve&db=Nucleotide&list_uids=90985494&dopt=GenBank&RID=AU1U5ZE6016&log$=nucltop&blast_rank=2) EST63066 Larval Stage 1 Aedes aegypti cDNA clo...  [227](http://blast.ncbi.nlm.nih.gov/Blast.cgi#90985494%2390985494) 5e-58

[gb|EE996646.1|](http://www.ncbi.nlm.nih.gov/entrez/query.fcgi?cmd=Retrieve&db=Nucleotide&list_uids=114944678&dopt=GenBank&RID=AU1U5ZE6016&log$=nucltop&blast_rank=3) EST68990 Larval Stage 1 Aedes aegypti cDNA clo...  [175](http://blast.ncbi.nlm.nih.gov/Blast.cgi#114944678%23114944678) 2e-42

[gb|DW994412.1|](http://www.ncbi.nlm.nih.gov/entrez/query.fcgi?cmd=Retrieve&db=Nucleotide&list_uids=85854452&dopt=GenBank&RID=AU1U5ZE6016&log$=nucltop&blast_rank=4) EST46793 Larval Stage 1 Aedes aegypti cDNA clo...  [171](http://blast.ncbi.nlm.nih.gov/Blast.cgi#85854452%2385854452) 3e-41

[gb|EE994584.1|](http://www.ncbi.nlm.nih.gov/entrez/query.fcgi?cmd=Retrieve&db=Nucleotide&list_uids=114938668&dopt=GenBank&RID=AU1U5ZE6016&log$=nucltop&blast_rank=5) EST67131 Larval Stage 1 Aedes aegypti cDNA clo...  [167](http://blast.ncbi.nlm.nih.gov/Blast.cgi#114938668%23114938668) 5e-40

[gb|CK726280.1|](http://www.ncbi.nlm.nih.gov/entrez/query.fcgi?cmd=Retrieve&db=Nucleotide&list_uids=42579818&dopt=GenBank&RID=AU1U5ZE6016&log$=nucltop&blast_rank=6) SWWbL3CAW12B10SK Wuchereria bancrofti L3 cDNA ...  [163](http://blast.ncbi.nlm.nih.gov/Blast.cgi#42579818%2342579818) 9e-39

[gb|EE996222.1|](http://www.ncbi.nlm.nih.gov/entrez/query.fcgi?cmd=Retrieve&db=Nucleotide&list_uids=114943819&dopt=GenBank&RID=AU1U5ZE6016&log$=nucltop&blast_rank=7) EST68607 Larval Stage 1 Aedes aegypti cDNA clo...  [153](http://blast.ncbi.nlm.nih.gov/Blast.cgi#114943819%23114943819) 7e-36

[gb|DW209572.1|](http://www.ncbi.nlm.nih.gov/entrez/query.fcgi?cmd=Retrieve&db=Nucleotide&list_uids=84265704&dopt=GenBank&RID=AU1U5ZE6016&log$=nucltop&blast_rank=8) EST25836 Larval Stage 1 Aedes aegypti cDNA clo...  [138](http://blast.ncbi.nlm.nih.gov/Blast.cgi#84265704%2384265704) 2e-31

[dbj|BJ630348.1|](http://www.ncbi.nlm.nih.gov/entrez/query.fcgi?cmd=Retrieve&db=Nucleotide&list_uids=37279574&dopt=GenBank&RID=AU1U5ZE6016&log$=nucltop&blast_rank=9) BJ630348 NIBB Mochii normalized Xenopus early...  [130](http://blast.ncbi.nlm.nih.gov/Blast.cgi#37279574%2337279574) 9e-29

[dbj|BJ613967.1|](http://www.ncbi.nlm.nih.gov/entrez/query.fcgi?cmd=Retrieve&db=Nucleotide&list_uids=37247109&dopt=GenBank&RID=AU1U5ZE6016&log$=nucltop&blast_rank=10) BJ613967 NIBB Mochii normalized Xenopus early...  [118](http://blast.ncbi.nlm.nih.gov/Blast.cgi#37247109%2337247109) 3e-25

[dbj|BJ630612.1|](http://www.ncbi.nlm.nih.gov/entrez/query.fcgi?cmd=Retrieve&db=Nucleotide&list_uids=37280104&dopt=GenBank&RID=AU1U5ZE6016&log$=nucltop&blast_rank=11) BJ630612 NIBB Mochii normalized Xenopus early... [92.8](http://blast.ncbi.nlm.nih.gov/Blast.cgi#37280104%2337280104) 4e-25

[gb|DW192348.1|](http://www.ncbi.nlm.nih.gov/entrez/query.fcgi?cmd=Retrieve&db=Nucleotide&list_uids=84232560&dopt=GenBank&RID=AU1U5ZE6016&log$=nucltop&blast_rank=12) EST08618 Larval Stage 1 Aedes aegypti cDNA clo...  [107](http://blast.ncbi.nlm.nih.gov/Blast.cgi#84232560%2384232560) 6e-22

[gb|EB097953.1|](http://www.ncbi.nlm.nih.gov/entrez/query.fcgi?cmd=Retrieve&db=Nucleotide&list_uids=90982813&dopt=GenBank&RID=AU1U5ZE6016&log$=nucltop&blast_rank=13) EST60385 Larval Stage 1 Aedes aegypti cDNA clo... [89.4](http://blast.ncbi.nlm.nih.gov/Blast.cgi#90982813%2390982813) 1e-16

[gb|EE994850.1|](http://www.ncbi.nlm.nih.gov/entrez/query.fcgi?cmd=Retrieve&db=Nucleotide&list_uids=114939190&dopt=GenBank&RID=AU1U5ZE6016&log$=nucltop&blast_rank=14) EST67371 Larval Stage 1 Aedes aegypti cDNA clo... [89.4](http://blast.ncbi.nlm.nih.gov/Blast.cgi#114939190%23114939190) 2e-16

[dbj|BJ613730.1|](http://www.ncbi.nlm.nih.gov/entrez/query.fcgi?cmd=Retrieve&db=Nucleotide&list_uids=37246630&dopt=GenBank&RID=AU1U5ZE6016&log$=nucltop&blast_rank=15) BJ613730 NIBB Mochii normalized Xenopus early... [79.3](http://blast.ncbi.nlm.nih.gov/Blast.cgi#37246630%2337246630) 2e-13

[gb|DW207317.1|](http://www.ncbi.nlm.nih.gov/entrez/query.fcgi?cmd=Retrieve&db=Nucleotide&list_uids=84261228&dopt=GenBank&RID=AU1U5ZE6016&log$=nucltop&blast_rank=16) EST23581 Larval Stage 1 Aedes aegypti cDNA clo... [74.7](http://blast.ncbi.nlm.nih.gov/Blast.cgi#84261228%2384261228) 4e-12

[gb|DW209989.1|](http://www.ncbi.nlm.nih.gov/entrez/query.fcgi?cmd=Retrieve&db=Nucleotide&list_uids=84266536&dopt=GenBank&RID=AU1U5ZE6016&log$=nucltop&blast_rank=17) EST26253 Larval Stage 1 Aedes aegypti cDNA clo... [62.8](http://blast.ncbi.nlm.nih.gov/Blast.cgi#84266536%2384266536) 2e-08

[gb|EB096938.1|](http://www.ncbi.nlm.nih.gov/entrez/query.fcgi?cmd=Retrieve&db=Nucleotide&list_uids=90981796&dopt=GenBank&RID=AU1U5ZE6016&log$=nucltop&blast_rank=18) EST59370 Larval Stage 1 Aedes aegypti cDNA clo... [53.1](http://blast.ncbi.nlm.nih.gov/Blast.cgi#90981796%2390981796) 1e-05

[gb|EG004580.1|](http://www.ncbi.nlm.nih.gov/entrez/query.fcgi?cmd=Retrieve&db=Nucleotide&list_uids=114972357&dopt=GenBank&RID=AU1U5ZE6016&log$=nucltop&blast_rank=19) EST76762 Larval Stage 1 Aedes aegypti cDNA clo... [52.0](http://blast.ncbi.nlm.nih.gov/Blast.cgi#114972357%23114972357) 3e-05

[gb|EE994665.1|](http://www.ncbi.nlm.nih.gov/entrez/query.fcgi?cmd=Retrieve&db=Nucleotide&list_uids=114938826&dopt=GenBank&RID=AU1U5ZE6016&log$=nucltop&blast_rank=20) EST67204 Larval Stage 1 Aedes aegypti cDNA clo... [40.4](http://blast.ncbi.nlm.nih.gov/Blast.cgi#114938826%23114938826) 0.091

[gb|DW990753.1|](http://www.ncbi.nlm.nih.gov/entrez/query.fcgi?cmd=Retrieve&db=Nucleotide&list_uids=85850793&dopt=GenBank&RID=AU1U5ZE6016&log$=nucltop&blast_rank=21) EST43134 Larval Stage 1 Aedes aegypti cDNA clo... [38.1](http://blast.ncbi.nlm.nih.gov/Blast.cgi#85850793%2385850793) 0.49

**Table S2:**  Overview of sequences encoding single (S)- and double (D)-domain EULs used to construct a phylogenetic tree (Figure 6). Sequences for all EUL proteins are shown in Additional file 1: Figure S5. v: vacuolar EUL homologs.

| Species | Code | Accession number/Locus/NCBI entries |
| --- | --- | --- |
| *Arabidopis thaliana* | ArathEULS1 | AF411801 |
| *Carica papaya* | CarpaEULS3 | ABIM01011929, LG3 contig (5531-7363) |
| *Euonymus europaeus* | EuoeuEULS0 | EF990655 |
| *Hordeum vulgare* | HorvuEULS1  HorvuEULS3  HorvuEULSv1  HorvuEULD1A  HorvuEULD1B | cDNA clone EBro02_SQ004_E24  cDNA clones HVSMEc0009K15f , HVSMEg0003G13f  and HO07I05  cDNA clone EBro08_SQ010_H21  cDNA clones MPMGp2010O246 and MPMGp2010P102  cDNA clones HC113B06_SK.ab1 and EBem09_SQ004_H21 |
| *Lactuca perennis* | LacpeEULS2  LacpeEULS3A | cDNA clone CLPX7549  cDNA clone CLPY10199 |
| *Lactuca saligna* | LacslEULS0  LacslEULS2 | cDNA clone CLLZ3957  cDNA clone CLLY9513 |
| *Lactuca sativa* | LacsaEULS2  LacsaEULS3A | cDNA clone CLSY3266  cDNA clone CLSX9689 |
| *Lactuca serriola* | LacseEULS0  LacseEULS2  LacseEULS3A | cDNA clone QGF17K08  cDNA clone CLRY3883  cDNA clone CLRX7784 |
| *Lactuca virosa* | LacviEULS0  LacviEULS2 | cDNA clone CLVY1312  cDNA clone CLVX7067 |
| *Marchantia polymorpha* | MarpoEULSm1  MarpoEULSm2 | complete cDNA clone lwa30n08  contig of cDNA clones lwa31d03 and lwb39k22 |
| *Medicago truncatula* | MedtrEULS3 | [gi|33186931|gb|AC145753.1|](http://www.ncbi.nlm.nih.gov/entrez/query.fcgi?cmd=Retrieve&db=Nucleotide&list_uids=33186931&dopt=GenBank) |
| *Oryza sativa* | OrysaEULS2  OrysaEULS3  OrysaEULD1A  OrysaEULD1B  OrysaEULD2 | Os07g0684000 (CAA70175)  Os01g0104400 (BAB16331)  Os07g0683900 (CAA70174)  Os03g0327600 (CAA64683)  Os07g0683600 (EAZ41120; sequence is incorrect) |
| *Physcomitrella patens* | PhypaEULS1  PhypaEULS3A  PhypaEULS3B  PhypaEULD1 | JGI: [scaffold_47](http://genome.jgi-psf.org/cgi-bin/getScaffold?db=Phypa1_1&scaffold=scaffold_47) (1265314- 1265892)  JGI: [scaffold_74](http://genome.jgi-psf.org/cgi-bin/getScaffold?db=Phypa1_1&scaffold=scaffold_74) ([415096:416617)](http://genome.jgi-psf.org/cgi-bin/getScaffold?db=Phypa1_1&scaffold=scaffold_74&start=415241&end=416617&sfStarts=415241,415629,415995,416354,&sfEnds=415477,415757,416216,416617,)  JGI: [scaffold_5](http://genome.jgi-psf.org/cgi-bin/getScaffold?db=Phypa1_1&scaffold=scaffold_5)| complement (2258832-2260516)  JGI: [scaffold_6](http://genome.jgi-psf.org/cgi-bin/getScaffold?db=Phypa1_1&scaffold=scaffold_6) complement (2986876 – 2989520) |
| *Picea sitchensis* | PicsiS2B  PicsiS2C  PicsiEULS3A  PicsiEULS3B  PicsiEULD1  PicsiEULD2 | cDNA clone WS02753_E16  cDNA clone WS0292_C20  cDNA clone WS0285_O02  cDNA clone WS02747_D08  cDNA clone WS02917_M08  cDNA clone WS0272_G09 |
| *Pinus taeda* | PintaEULS2A  PintaEULS2B  PintaEULS3A  PintaEULS3B  PintaEULD1  PintaEULD2A  PintaEULD2B | cDNA clone RTBOR1_25_E05_A029  cDNA clone NDL1_56_D05_A029  cDNA clone RTFE1_44_G06_A029  cDNA clone FLD1_55_D05_A029  cDNA clone RTFE1_5_H06_A029  cDNA clones RTFEPL1_31_B04_A029,  RTCNT2_1_D06_A029 and RTFE1_47_B09_A029  cDNA clones RTBOR1_11_F02_A029 and  RTFE1_8_G12_A029 |
| *Plantago major* | PlamaEULS0A | CAH59433, contig of EX266294 |
| *Populus trichocarpa* | PoptrEULS3A  PoptrEULS3B | >[LG_XIX](http://genome.jgi-psf.org/cgi-bin/getScaffold?db=Poptr1&scaffold=LG_XIX)|join (4709049 -4709669; 4710089- 4710211;  4711609 -4711843; 4712032-4712033) (ABK95474.1)  >[LG_XIII](http://genome.jgi-psf.org/cgi-bin/getScaffold?db=Poptr1&scaffold=LG_XIII) join (3942835-3943566 3944069 – 3944191;  3947672-3947906; 3948114-3948114)  (No NCBI annotation) |
| *Ricinus communis* | RiccoEULS3 | >rca1.assembly.29950 Join complement (317119-317120,  317440-317668, 318586- 318708, 319075- 319635) |
| *Selaginella moellendorffi* | SelmoEULS0  SelmoEULS1A  SelmoEULS1B  SelmoEULS1C  SelmoEULS3  SelmoEULS4  SelmoEULSv1  SelmoEULSv2  SelmoEULD0  SelmoEULD3  SelmoEULD4A  SelmoEULD4B  SelmoEULD4C  SelmoEULD4D  SelmoEULD4E  SelmoEULD4F | >gnl|Nov06_contig|383.1 Complement (24747-25434)  >gnl|Nov06_contig|383.1 (26989-27622)  >[gnl|Nov06_contig|161.14](http://selaginella.genomics.purdue.edu/cgi-bin/blast_basic_s.cgi?cmd=fastacmd&datafile=Nov06_contig&uid=161.14) (4841-5502)  >[gnl|Nov06_contig|539.1](http://selaginella.genomics.purdue.edu/cgi-bin/blast_basic_s.cgi?cmd=fastacmd&datafile=Nov06_contig&uid=539.1) Complement (7840-8510)  >[gnl|Nov06_contig|41.13](http://selaginella.genomics.purdue.edu/cgi-bin/blast_basic_s.cgi?cmd=fastacmd&datafile=Nov06_contig&uid=41.13) (35126 -36003)  >[gnl|Nov06_contig|41.13](http://selaginella.genomics.purdue.edu/cgi-bin/blast_basic_s.cgi?cmd=fastacmd&datafile=Nov06_contig&uid=41.13) (36308- 37316)  >[gnl|Nov06_contig|207.9](http://selaginella.genomics.purdue.edu/cgi-bin/blast_basic_s.cgi?cmd=fastacmd&datafile=Nov06_contig&uid=207.9) Complement (10001-10531)  >[gnl|Nov06_contig|207.9](http://selaginella.genomics.purdue.edu/cgi-bin/blast_basic_s.cgi?cmd=fastacmd&datafile=Nov06_contig&uid=207.9) Complement (8947-9486)  >gnl|Nov06_contig|383.1 (28214-29554)  >gnl|Nov06_contig|383.1 (31232 - 32889)  >[gnl|Nov06_contig|19.12](http://selaginella.genomics.purdue.edu/cgi-bin/blast_basic_s.cgi?cmd=fastacmd&datafile=Nov06_contig&uid=19.12) complement (102686-104014)  >[gnl|Nov06_contig|37.7](http://selaginella.genomics.purdue.edu/cgi-bin/blast_basic_s.cgi?cmd=fastacmd&datafile=Nov06_contig&uid=37.7) complement (9708-10994)  >[gnl|Nov06_contig|37.7](http://selaginella.genomics.purdue.edu/cgi-bin/blast_basic_s.cgi?cmd=fastacmd&datafile=Nov06_contig&uid=37.7) complement (7429-8702)  >[gnl|Nov06_contig|37.12](http://selaginella.genomics.purdue.edu/cgi-bin/blast_basic_s.cgi?cmd=fastacmd&datafile=Nov06_contig&uid=37.12) Complement (8599-9885)  >[gnl|Nov06_contig|37.16](http://selaginella.genomics.purdue.edu/cgi-bin/blast_basic_s.cgi?cmd=fastacmd&datafile=Nov06_contig&uid=37.16) complement (3756-5068)  >[gnl|Nov06_contig|659.5](http://selaginella.genomics.purdue.edu/cgi-bin/blast_basic_s.cgi?cmd=fastacmd&datafile=Nov06_contig&uid=659.5) Complement (1640-2926) |
| *Sorghum bicolor* | SorbiEULS1  SorbiEULS3  SorbiEULSv1  SorbiEULSv2  SorbiEULSv3  SorbiEULD1A  SorbiEULD1B  SorbiEULD1C  SorbiEULD2 | cDNA clone GABR1_52_C01_A002  cDNA clones OV1_22_A04.b1_A002,  DSAF1_72_G07_A011 and DSAF1_79_G06_A011  cDNA clone ANR1_20_C08_A002  cDNA clone ANR1_21_D02_A002  super_147 complement (720443-721526)  cDNA clones PH1_10_H09_A002 and  RHOH1_35_E11_A002  cDNA clone RHOH1_14_B05_A002  cDNA clone SS1_3_E05_A012 completed  with NCBI Trace archives sequences  cDNA clone OX1_10_C06_A002 |
| *Triticum aestivum* | TriaeEULS1A  TriaeEULS3A  TriaeEULSv1  TriaeEULD1A  TriaeEULD1B | cDNA clone whv3n4l14  cDNA clones TaLr1162C05R, TaE05030H08F,  MUG020.B08 and whsl23b17  cDNA clones whr2n09 and WHE1216_F12_L24  cDNAclones FGAS029769 and WHE0425_E02_I03  cDNA clone whyd20a08 |
| *Vitis* *vinifera* | VitviEULS3 | [gi|110720494|gb|EE096464.1|](http://www.ncbi.nlm.nih.gov/entrez/query.fcgi?cmd=Retrieve&db=Nucleotide&list_uids=110720494&dopt=GenBank) |
| *Zea mays* | ZeamaEULS0  ZeamaEULS2  ZeamaEULS3A  ZeamaEULSv1  ZeamaEULD1A  ZeamaEULD1B  ZeamaEULD2 | cDNA zmrww00_0B20-009-b10.s1  cDNA clone ZM_BFc0163K11  cDNA clone ZM_BFc0109O11  cDNA clone QBS2f12  cDNA clone ZM_BFc0029N11  cDNA clone ZM_BFc0171G10  cDNA clones ZM_BFb0279G10.r, ZM_BFc0034L13 and ZM_BFb0162I07.f |

* *Selaginella* sequences are not annotated yet, but have been taken from the genomic sequences deposited in the *Selaginella* database (http://selaginella.genomics.purdue.edu/).

** *Sorghum* genomic sequences are from the Phytozome annotation.
